# Genotype × Year Interaction Dominates Mineral Variation in Berry Fractions of Eight *Vitis labrusca* Cultivars

**DOI:** 10.1002/fsn3.72438

**Published:** 2026-10-03

**Authors:** Besim Karabulut, Yahya Uray, Hüseyin Çelik, Bülent Köse, Cafer Kose, Metin Turan, Ozkan Kaya

**Affiliations:** ^1^ Department of Horticulture, Faculty of Agriculture Ondokuz Mayıs University Samsun Türkiye; ^2^ Department of Horticulture, Faculty of Agriculture Ataturk University Erzurum Türkiye; ^3^ Faculty of Economy and Administrative Science Yeditepe University Istanbul Türkiye; ^4^ Department of Life Sciences Western Caspian University Baku Azerbaijan; ^5^ Department of Horticulture and Agronomy, Faculty of Agriculture Kyrgyz‐Turkish Manas University Bishkek Kyrgyzstan; ^6^ Republic of Türkiye Ministry of Agriculture and Forestry General Directorate of Agricultural Research and Policies, Erzincan Horticultural Research Institute Erzincan Türkiye

**Keywords:** berry fractionation, compositional stability, genotype × environment interaction, mineral composition, variance partitioning, *Vitis labrusca*

## Abstract

The mineral composition of grape berries is shaped simultaneously by genotype and by growing season, yet the relative contribution of each source, and of their interaction, is rarely quantified within a single experimental framework. We determined 13 macro‐ and micronutrients (N, P, K, Ca, Mg, S, Mn, Fe, Zn, B, Cu, Na, and Cl) in four berry fractions (seed, skin, pulp, and juice) of eight *Vitis labrusca* cultivars (Concord, Niagara, Steuben, Rizessi, Rizpem, Rizellim, Ülkemiz, and Çeliksu) grown under a uniform, nonirrigated, and non‐fertilized regime at a single site over two consecutive growing seasons (2024 and 2025). Data were analyzed within each fraction using a factorial model containing block, year, cultivar, and the year × cultivar interaction, with false‐discovery‐rate correction applied across all tests. The year × cultivar interaction was significant for 49 of 52 fraction × element combinations and accounted for the largest single share of total variance (mean 39%), exceeding the contributions of cultivar (32%) and year (14%); the interaction was strongest in the pulp (43%) and juice (44%) fractions and reached 74% for pulp manganese. Cultivar effects within a season were significant in 93 of 104 tests. Interannual change was directionally coherent for several elements: all 16 significant changes in iron and all 20 in zinc were increases in 2025, whereas 17 of 19 significant manganese changes and 13 of 14 phosphorus changes were decreases. Cultivars differed substantially in compositional stability between seasons, with Niagara varying least (median 1.75‐fold change across elements and fractions) and Concord most (2.87‐fold); Niagara and Rizpem were particularly stable in the pulp and juice. Harvest maturity, expressed as soluble solids concentration, was not associated with mineral concentration in any fraction. After cultivar, year, and block effects were removed, 186 of 312 elemental pairs remained significantly correlated, indicating associations that are not merely a reflection of cultivar or seasonal differences. These findings identify genotype × environment interaction, rather than genotype alone, as the principal determinant of mineral variation in *V. labrusca* berries and suggest that compositional stability is itself a trait that differs among cultivars. Absolute concentrations reported here should be regarded as provisional, because analytical validation data for the present dataset were not available.

## Graphical abstract

Thirteen macro‐ and micronutrients were quantified in the seed, skin, pulp and juice of eight *Vitis labrusca* cultivars grown under uniform, nonirrigated and non‐fertilized conditions over two seasons (2024–2025). The year × cultivar interaction was significant for 49 of 52 fraction × element combinations and explained the largest share of variance (mean 39%), exceeding cultivar (32%) and year (14%) effects, with the strongest interactions in pulp and juice. Iron and zinc consistently increased in 2025, whereas manganese and phosphorus decreased. Cultivars differed in compositional stability, with Niagara most stable and Concord least. Genotype × environment interaction, not genotype alone, is the principal driver of mineral variation in *V. labrusca* berries.

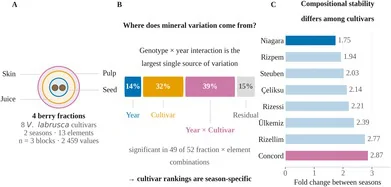

## 1 Introduction

Mineral elements are indispensable constituents of plant metabolism, playing critical roles in enzymatic activity, osmotic regulation, structural integrity, and the biosynthesis of essential biomolecules (Bertoldi et al. 2011). These elements are not only vital for plant growth and development, but their accumulation and distribution across different tissue compartments also directly reflect soil chemistry, root uptake efficiency, and phloem and xylem mobility patterns within the plant (Rogiers et al. 2006; Bertoldi et al. 2011). In this context, fruit crops represent particularly important sinks for mineral elements, as their diverse anatomical fractions (skin, pulp, seed, and juice) differ substantially in their capacity to retain and concentrate specific nutrients (Marin et al. 2009). Among fruit crops, grapes (*Vitis* spp.) occupy a position of exceptional significance, ranking as the fourth largest fruit crop globally with an annual production exceeding 77 million tons and being cultivated across temperate, Mediterranean, and subtropical regions for fresh consumption and for the manufacture of wine, juice, raisins, and a wide range of derived products (FAO 2021; Colak and Bengu 2024). Beyond their economic importance, grape berries are recognized as a valuable dietary source of macronutrients, including potassium, calcium, magnesium, and phosphorus, as well as micronutrients such as iron, zinc, manganese, and boron, all of which make a meaningful contribution to human nutritional security (Marin et al. 2009; Székelyhidi et al. 2024). Given these nutritional attributes, understanding the elemental composition of grape berries across their distinct anatomical fractions is therefore of fundamental importance not only for plant physiology but also for food quality assessment.

The mineral composition of grape berries is not uniform across anatomical fractions; rather, it varies markedly among seeds, skin, pulp, and juice depending on the mobility of each element within the vascular system. Rogiers et al. (2006) demonstrated that phloem‐mobile elements such as boron, copper, iron, potassium, magnesium, phosphorus, and sulfur accumulate progressively throughout berry development, whereas xylem‐mobile minerals including calcium, manganese, and zinc reach their maximum concentrations prior to *véraison* and are preferentially partitioned into seeds. Bertoldi et al. (2011) further confirmed these partitioning patterns in *Vitis vinifera* cv. Chardonnay, documenting fraction‐specific enrichment for 55 macro‐, micro‐, and trace elements across berry development. Similarly, Tangolar et al. (2009, 2020) reported significant genotypic variation in mineral content among wine grape cultivars, while Gomes et al. (2019) highlighted that the bioavailability of minerals is strongly influenced by grape genotype and prevailing environmental conditions. These findings collectively underscore that a single whole‐berry analysis is insufficient and that fraction‐specific characterization is necessary to fully capture the nutritional and biochemical significance of grape mineral composition.

Cultivar identity constitutes one of the most influential determinants of mineral accumulation in grape berries. Significant inter‐cultivar variation in macronutrient and micronutrient concentrations has been documented across diverse genetic backgrounds, including European *V. vinifera* varieties and their interspecific hybrids (Al Juhaimi et al. 2017; Özcan et al. 2017; Tangolar et al. 2020). Székelyhidi et al. (2024) demonstrated that micro‐ and macroelement contents differ substantially among eight cultivars grown under identical management conditions, with copper, iron, and zinc exhibiting the greatest inter‐cultivar variability. Furthermore, year‐to‐year climatic fluctuations introduce an additional source of variation that is rarely addressed simultaneously with cultivar effects in a multi‐fraction analytical framework (Colak and Bengu 2024). Despite growing interest in the nutritional characterization of grapes and their by‐products, particularly seeds and skins as sources of bioactive compounds and minerals for the food industry (Mironeasa et al. 2010; Hatterman‐Valenti et al. 2024; Haider et al. 2025; Amjad et al. 2026), systematic, multi‐fraction, multi‐year data comparing cultivars under uniform growing conditions remain scarce, particularly for aromatic non‐vinifera species. A further limitation of the existing literature is that cultivar and year effects are most often reported as separate main effects, so that the extent to which cultivars respond differently to the same season, that is, the genotype × environment interaction, remains largely unquantified for grape mineral composition. This distinction is not merely statistical. If the interaction term is small, a single‐season survey provides a reasonable description of a cultivar's mineral profile and cultivars can be ranked reliably. If the interaction term is large, cultivar rankings are season‐specific, single‐year characterizations are misleading, and the stability of a cultivar's composition across seasons becomes a trait in its own right. Partitioning the total variance into year, cultivar and interaction components offers a direct means of resolving this question, but such partitioning has not been reported for berry fractions of any *Vitis* species.

*Vitis labrusca* L. and its hybrids, including well‐known cultivars such as Concord and Niagara as well as locally bred Turkish foxy cultivars, represent a group of grapevines of increasing agricultural and nutritional interest, particularly in humid regions where *V. vinifera* cultivation is constrained by fungal disease pressure (Keskin et al. 2021; Colak and Bengu 2024). Despite their widespread cultivation in the Black Sea region of Turkey and their high production potential, the mineral composition of *V. labrusca* cultivars across distinct berry fractions has received far less attention than their *V. vinifera* counterparts. The present study was therefore designed to address this gap by investigating thirteen mineral elements across four berry fractions in eight *V. labrusca* cultivars over two consecutive growing seasons (2024–2025) under a uniform, nonirrigated, non‐fertilized management regime. In this regard, the specific objectives of our study were: (i) to quantify inter‐cultivar variation in mineral content within each berry fraction and within each season; (ii) to characterize the magnitude and direction of interannual change for each element; (iii) to partition the total variance of each fraction × element combination into year, cultivar and year × cultivar components, and to rank cultivars according to the stability of their mineral composition between seasons; and (iv) to examine associations among mineral elements and the multivariate structure of mineral composition after cultivar and year effects have been accounted for.

## 2 Materials and Methods

### 2.1 Plant Material, Experimental Site, and Experimental Design

This study was conducted during the 2024 and 2025 growing seasons at the Research Vineyard of the Faculty of Agriculture, Ondokuz Mayıs University, Samsun, Türkiye (41.364295° N, 36.191687° E). Eight *Vitis labrusca* L. cultivars were used as plant material: Concord, Niagara, and Steuben, which are well‐established North American cultivars widely grown for juice and wine production; and Rizessi, Rizpem, Rizellim, Ülkemiz, and Çeliksu, which are Turkish‐bred foxy grape cultivars developed for local production conditions (Figure 1). All vines were own‐rooted, 10 years old, and trained to a high‐trunk bilateral cordon system at a planting distance of 3.0 × 1.5 m. Dormant pruning was applied as spur pruning to two to three buds per spur to maintain a uniform bud load across all cultivars. No irrigation and no fertilization were applied at any point during the experimental period, and no copper‐based or other plant‐protection product was applied to the vineyard in either season; the vines were managed under natural growing conditions throughout. The experiment was established in a randomized complete block design (RCBD) with three blocks (replications), each block comprising six vines per cultivar. Within each block, the five clusters collected from the six vines of a given cultivar were combined into a single composite sample prior to fractionation and analysis. The experimental unit is therefore the block‐level composite sample, giving *n* = 3 biological replicates per cultivar and season; no technical replicates were analyzed, and the three values reported for each cultivar × year × fraction combination represent three independent field replicates. Block was retained as a factor in all statistical models (Section 2.4). The same vines were followed in both seasons.

**FIGURE 1:**
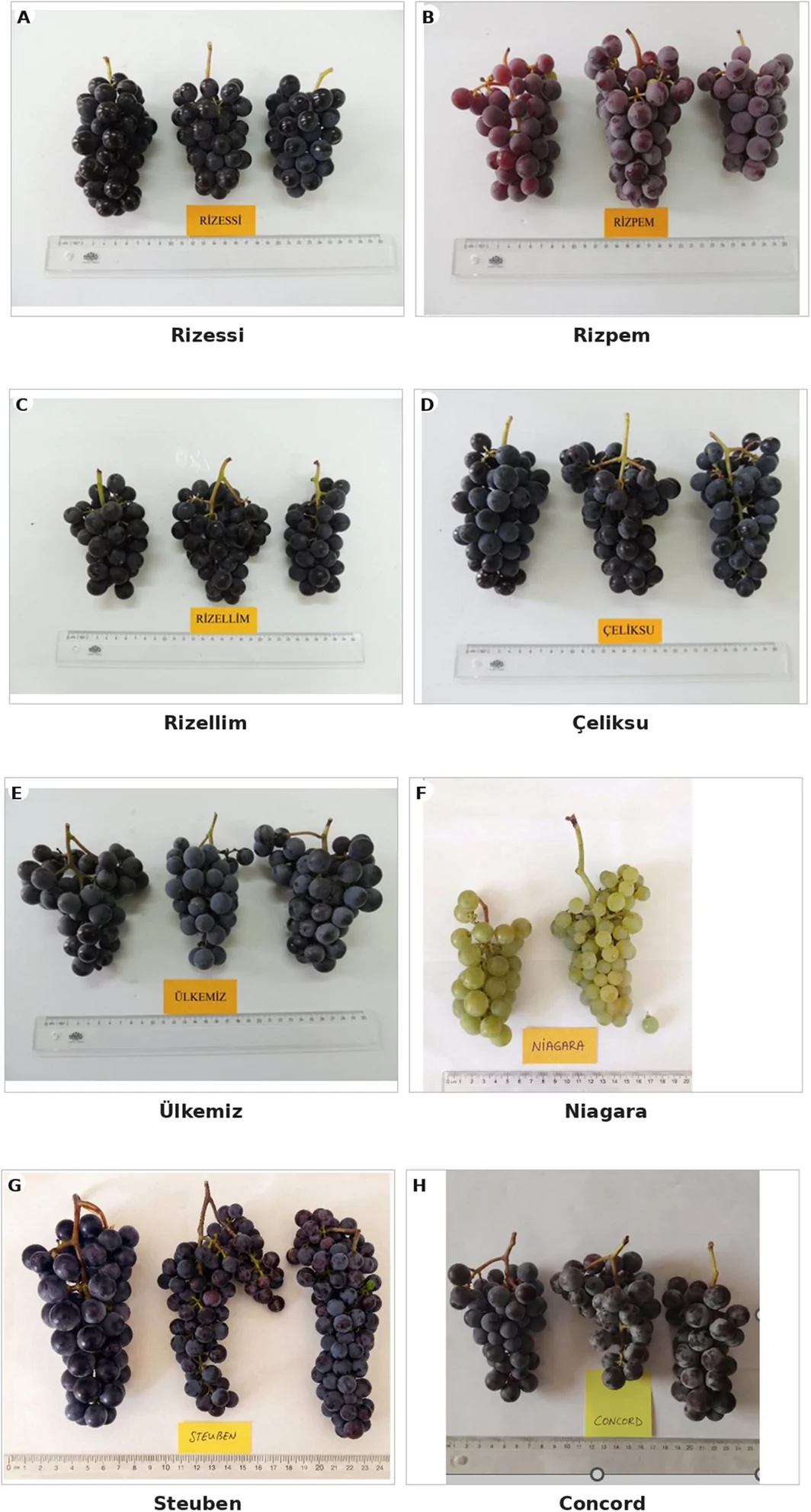
Representative bunch photographs of the eight *Vitis labrusca* cultivars used in the study: (A) Rizessi, (B) Rizpem, (C) Rizellim, (D) Çeliksu, (E) Ülkemiz, (F) Niagara, (G) Steuben, and (H) Concord.

### 2.2 Harvest and Sample Preparation

Harvest was performed when berry soluble solids concentration (SSC) reached approximately 18–22 °Brix, as assessed using a hand refractometer (ATAGO, Japan). Harvest dates and the SSC recorded for each cultivar in each season are given in Table S2. Harvest in 2025 took place on average 7.1 days later than in 2024, and SSC ranged from 17.6 to 22.8 °Brix across cultivars and seasons. Because this range spans approximately five degrees Brix and mineral accumulation is known to depend on developmental stage, SSC was tested explicitly as a covariate at the cultivar × year level (Section 2.4). At each harvest date, five representative clusters per block were collected. In the laboratory, berries were selected from the basal, middle, and apical portions of each cluster to ensure representative sampling. Berries were then manually fractionated into four anatomical components: seed, berry skin, berry pulp, and juice. Juice was obtained by gently pressing the pulp fraction through a nylon mesh. All fractions were immediately frozen and stored at −20°C until analysis.

### 2.3 Mineral Analysis

Prior to analysis, seed, skin, and pulp fractions were oven‐dried at 65°C to constant weight and subsequently ground to a homogeneous fine powder using a laboratory mill. Approximately 0.5 g of each dried fraction was subjected to microwave‐assisted acid digestion (CEM Mars 6, Matthews, NC, USA) in a closed‐vessel system using a mixture of concentrated HNO₃ and H_2_O_2_ (4:1, v/v) at 180°C for 20 min, following the procedure described by AOAC (2016). Juice samples were digested directly, without prior drying, using the same acid mixture and digestion program. Eleven elements, namely phosphorus (P), potassium (K), calcium (Ca), magnesium (Mg), sulfur (S), manganese (Mn), iron (Fe), zinc (Zn), boron (B), copper (Cu), and sodium (Na), were determined in the digested solutions, which were filtered and made up to volume with ultrapure water (18.2 MΩ cm), by inductively coupled plasma optical emission spectrometry (ICP‐OES; Agilent 5110, Santa Clara, CA, USA). Total nitrogen (N) was not determined by ICP‐OES but by the Kjeldahl method, and chloride (Cl) was determined by ion chromatography on a separate aqueous extract rather than on the acid digest; both procedures followed the reference methods compiled by Kalra (1998). Macronutrient concentrations (N, P, K, Ca, Mg, S) are expressed as a percentage of dry weight (%), and micronutrient concentrations (Mn, Fe, Zn, B, Cu, Na, Cl) as milligrams per kilogram of dry weight (mg kg^−1^).

Certified reference materials and procedural blanks were included in each analytical batch for quality assurance. However, method‐performance parameters, namely limits of detection and quantification, spike recovery, certified‐reference‐material recovery and calibration linearity, were not systematically recorded for the present dataset and therefore cannot be reported here. The variability among the three field replicates, expressed as the relative standard deviation, ranged from 9.4% for N to 24.2% for B; this figure combines analytical and biological variation and is reported as replicate variability rather than as a measure of analytical precision. In addition, because the juice fraction was digested without prior drying and the procedure used to convert the measured concentrations to a dry‐weight basis could not be documented retrospectively, the absolute concentrations reported for this fraction, and by extension the direct comparison of concentrations between fractions, cannot be verified. For this reason, all absolute values in this study are regarded as provisional, no comparison is made between berry fractions, and all interpretations are restricted to comparisons among cultivars and between seasons within a single fraction. Such within‐fraction comparisons are unaffected by any constant scaling factor and are therefore valid irrespective of the conversion applied. These limitations are stated in full in Section 5.

### 2.4 Statistical Analysis

All analyses were performed in R 4.3.1 (R Core Team 2023) and Python 3.12; figures were produced with matplotlib (Hunter 2007). The dataset was first verified in full against the original laboratory records. Three categories of discrepancy were identified and are documented, with the action taken for each, in Table S1; 2459 of 2496 values (98.5%) were retained for analysis. Each fraction × element combination was analyzed separately with the factorial model log(*y*) ~ Block + Year × Cultivar, using Type II sums of squares. The log transformation was applied because it improved the normality of model residuals and because variance homogeneity across cultivar × year cells held on the log scale for all 52 combinations. Block was retained in every model irrespective of its significance, in order to reflect the blocking structure of the design in the estimation of the error term. Data from the two seasons were not pooled at any stage, because the year × cultivar interaction was significant for 49 of the 52 combinations (Section 3.4); cultivar means are therefore reported separately by season throughout, as mean ± standard deviation of the three field replicates. The total sum of squares of each model was partitioned into the proportions attributable to year, cultivar, the interaction and the residual (Table S3, Figure 2A). Because the interaction was significant almost throughout, cultivar was additionally evaluated as a simple effect within each season using log(*y*) ~ Block + Cultivar, with mean separation by Tukey's honestly significant difference test (α = 0.05) on the log scale. All *p* values were adjusted by the Benjamini–Hochberg false‐discovery‐rate procedure within each family of tests, and adjusted values are reported as *q* throughout. Interannual change was expressed for each cultivar × fraction × element combination as the log_2_ ratio of the 2025 to the 2024 mean and tested by Welch's *t*‐test on log‐transformed values; the compositional stability of each cultivar was summarized as the median absolute log_2_ fold change across all elements and fractions, a measure independent of the absolute concentration scale. Associations among elements were examined as partial correlations, computed among residuals after each element had been regressed on cultivar, year and block, because simple correlations across pooled observations may reflect differences among cultivars or seasons rather than relationships among elements. Principal component analysis was performed for each fraction on log‐transformed and standardized replicate‐level observations (*n* = 44–45) rather than on cultivar × year means. Finally, soluble solids concentration at harvest was tested as a covariate of mineral concentration at the cultivar × year level for every fraction × element combination.

**FIGURE 2:**
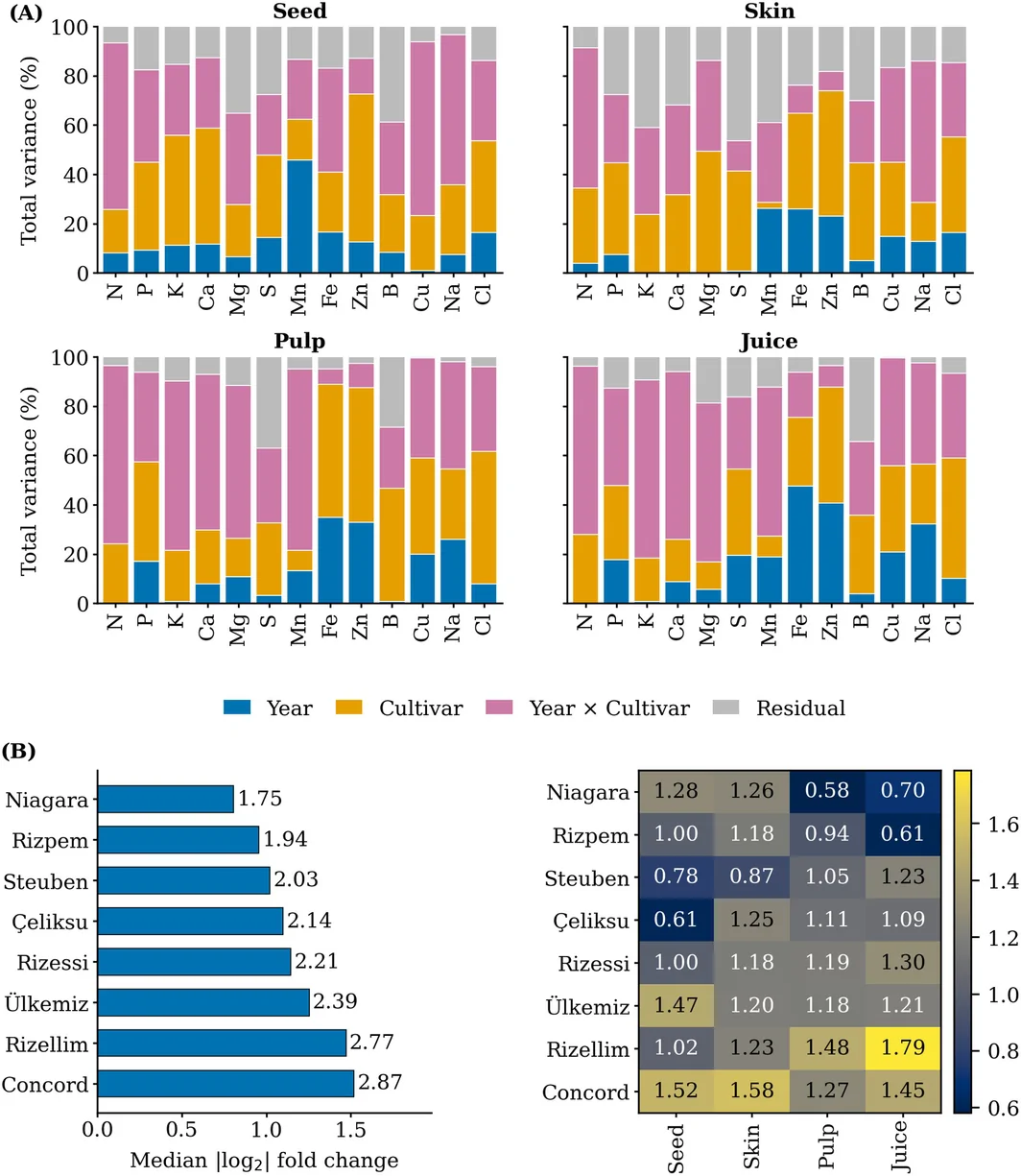
Sources of variation in mineral concentration and interannual stability of the eight cultivars. (A) Partitioning of the total variance of each fraction × element combination into year, cultivar, year × cultivar and residual components, derived from the model log(*y*) ~ Block + Year × Cultivar fitted separately for each fraction and element. (B) Interannual instability of mineral composition, expressed as the median absolute log_2_ fold change between the 2024 and 2025 seasons across all 13 elements (left panel, with the corresponding fold change given beside each bar) and resolved by berry fraction (right panel). Lower values indicate greater compositional stability between seasons. Both measures are ratios and are therefore independent of the absolute concentration scale.

## 3 Results

### 3.1 Macronutrient Concentrations Within Berry Fractions

Macronutrient concentrations for each cultivar, fraction and season are presented in Table 1 and illustrated in Figure 3. Because the year × cultivar interaction was significant for almost all combinations (Section 3.4), cultivar effects are reported as simple effects within each season. Across all four fractions and both seasons, the effect of cultivar on macronutrient concentration was significant in 44 of the 48 fraction × element × season combinations examined. In the seed fraction, nitrogen differed among cultivars in both seasons but with a complete reversal of ranking: Concord recorded the highest concentration in 2024 (1.08%) and the lowest in 2025 (0.37%), whereas Rizessi rose from 0.68% to the highest value of the panel in 2025 (1.47%). Potassium showed the widest inter‐cultivar range in 2024, from 0.890% in Ülkemiz to 0.143% in Steuben, but the spread narrowed considerably in 2025, when Çeliksu recorded the highest value (0.293%) and Steuben again the lowest (0.086%). Calcium followed a comparable pattern, with Concord highest in 2024 (0.727%) and Rizpem highest in 2025 (0.283%), Niagara recording the lowest value in both seasons. Phosphorus was highest in Concord in 2024 (0.240%) and in Steuben in 2025 (0.290%), and Niagara consistently recorded the lowest concentration in both seasons (0.070% and 0.037%). Magnesium differed among cultivars in both seasons (*q* < 0.05 and *q* < 0.01), and sulfur did not differ significantly in 2024 (*q* > 0.05) although it did in 2025, when Ülkemiz recorded the highest value (0.070%).

**TABLE 1:** Macronutrient concentrations (% of dry weight) in four berry fractions of eight *Vitis labrusca* cultivars in the 2024 and 2025 growing seasons.

Cultivar	N	P	K	Ca	Mg	S
Berry seed—2024						
Concord	1.08 ± 0.05 a	0.240 ± 0.020 a	0.40 ± 0.02 b	0.727 ± 0.055 a	0.0667 ± 0.0058 ab	0.0733 ± 0.0115 a
Çeliksu	0.48 ± 0.06 cde	0.076 ± 0.015 bc	0.20 ± 0.03 cd	0.140 ± 0.010 de	0.0300 ± 0.0100 ab	0.0633 ± 0.0153 a
Niagara	0.37 ± 0.04 e	0.070 ± 0.052 c	0.18 ± 0.06 cd	0.117 ± 0.021 e	0.0300 ± 0.0173 b	0.0467 ± 0.0115 a
Rizellim	0.96 ± 0.08 ab	0.100 ± 0.010 abc	0.32 ± 0.01 b	0.273 ± 0.015 bc	0.0700 ± 0.0100 a	0.0500 ± 0.0100 a
Rizessi	0.68 ± 0.18 bc	0.180 ± 0.020 ab	0.25 ± 0.01 bc	0.217 ± 0.035 cd	0.0467 ± 0.0115 ab	0.0367 ± 0.0058 a
Rizpem	0.84 ± 0.10 ab	0.173 ± 0.015 ab	0.19 ± 0.03 cd	0.203 ± 0.015 cd	0.0467 ± 0.0115 ab	0.0533 ± 0.0252 a
Steuben	0.42 ± 0.12 de	0.080 ± 0.052 bc	0.14 ± 0.01 d	0.150 ± 0.078 de	0.0300 ± 0.0173 b	0.0567 ± 0.0379 a
Ülkemiz	0.64 ± 0.06 bcd	0.177 ± 0.025 ab	0.89 ± 0.04 a	0.410 ± 0.026 b	0.0367 ± 0.0058 ab	n.d.
*F* (cultivar)	15.63***	6.65**	40.71***	29.34***	3.88*	1.09 ns
Berry seed—2025						
Concord	0.37 ± 0.04 b	0.056 ± 0.005 bc	0.18 ± 0.01 ab	0.140 ± 0.020 abc	0.0300 ± 0.0100 ab	0.0500 ± 0.0100 a
Çeliksu	1.33 ± 0.08 a	0.070 ± 0.017 bc	0.29 ± 0.03 a	0.180 ± 0.035 ab	0.0500 ± 0.0100 ab	0.0267 ± 0.0058 ab
Niagara	1.03 ± 0.19 a	0.0367 ± 0.0289 c	0.16 ± 0.16 ab	0.083 ± 0.075 c	0.0233 ± 0.0231 b	0.0100 ± 0.0100 b
Rizellim	0.48 ± 0.04 b	0.080 ± 0.010 b	0.23 ± 0.04 a	0.187 ± 0.031 ab	0.0333 ± 0.0058 ab	0.0533 ± 0.0115 a
Rizessi	1.47 ± 0.07 a	0.107 ± 0.015 b	0.25 ± 0.02 a	0.253 ± 0.021 a	0.0233 ± 0.0058 ab	0.0367 ± 0.0058 a
Rizpem	1.19 ± 0.16 a	0.083 ± 0.011 b	0.23 ± 0.01 a	0.283 ± 0.025 a	0.0233 ± 0.0058 ab	0.0133 ± 0.0058 ab
Steuben	1.21 ± 0.20 a	0.290 ± 0.036 a	0.08 ± 0.01 b	0.096 ± 0.005 bc	0.0700 ± 0.0100 a	0.0400 ± 0.0000 a
Ülkemiz	0.48 ± 0.11 b	0.076 ± 0.020 b	0.17 ± 0.02 ab	0.147 ± 0.006 abc	0.0333 ± 0.0153 ab	0.0700 ± 0.0200 a
*F* (cultivar)	80.22***	21.85***	5.14**	8.61***	4.71**	5.65**
Berry skin—2024						
Concord	1.77 ± 0.37 a	0.480 ± 0.113 a	1.14 ± 0.41 ab	1.030 ± 0.340 a	0.130 ± 0.053 ab	0.243 ± 0.091 a
Çeliksu	0.77 ± 0.04 c	0.177 ± 0.029 d	0.64 ± 0.11 b	0.230 ± 0.030 c	0.060 ± 0.010 c	0.200 ± 0.046 ab
Niagara	0.92 ± 0.03 c	0.603 ± 0.110 a	1.18 ± 0.07 ab	0.127 ± 0.006 d	0.046 ± 0.011 c	0.066 ± 0.037 c
Rizellim	1.69 ± 0.15 a	0.270 ± 0.000 bc	1.17 ± 0.06 ab	0.473 ± 0.021 b	0.203 ± 0.006 a	0.210 ± 0.035 ab
Rizessi	1.24 ± 0.10 b	0.517 ± 0.059 a	1.34 ± 0.11 a	0.440 ± 0.080 b	0.137 ± 0.015 ab	0.163 ± 0.023 ab
Rizpem	1.62 ± 0.04 ab	0.180 ± 0.000 d	0.31 ± 0.02 c	0.140 ± 0.000 d	0.056 ± 0.005 c	0.086 ± 0.047 bc
Steuben	0.89 ± 0.07 c	0.310 ± 0.026 b	1.03 ± 0.45 ab	0.343 ± 0.064 bc	0.110 ± 0.026 b	0.347 ± 0.038 a
Ülkemiz	0.55 ± 0.02 d	0.200 ± 0.010 cd	1.31 ± 0.47 a	0.377 ± 0.025 b	0.046 ± 0.005 c	n.d.
*F* (cultivar)	49.96***	42.05***	11.42***	51.68***	20.97***	10.44***
Berry skin—2025						
Concord	0.59 ± 0.07b	0.140 ± 0.010 b	0.69 ± 0.08 a	0.253 ± 0.042 a	0.0667 ± 0.0058 c	0.163 ± 0.015 a
Çeliksu	2.19 ± 0.26 a	0.240 ± 0.066 ab	1.59 ± 0.51 a	0.420 ± 0.130 a	0.1670 ± 0.0640 ab	0.130 ± 0.046 a
Niagara	2.46 ± 0.87 a	0.307 ± 0.232 ab	0.94 ± 0.60 a	0.407 ± 0.293 a	0.0567 ± 0.0252 c	0.160 ± 0.125 a
Rizellim	0.78 ± 0.17 b	0.157 ± 0.023 ab	0.67 ± 0.03 a	0.273 ± 0.045 a	0.0733 ± 0.0208 c	0.180 ± 0.035 a
Rizessi	2.81 ± 0.44 a	0.357 ± 0.081 ab	1.30 ± 0.23 a	0.643 ± 0.168 a	0.0867 ± 0.0252 bc	0.117 ± 0.015 a
Rizpem	1.01 ± 0.44 b	0.197 ± 0.040 ab	0.82 ± 0.23 a	0.347 ± 0.165 a	0.0733 ± 0.0058 bc	0.173 ± 0.116 a
Steuben	2.84 ± 0.90 a	0.643 ± 0.112 a	1.84 ± 1.36 a	0.547 ± 0.330 a	0.2330 ± 0.0510 a	0.190 ± 0.036 a
Ülkemiz	0.71 ± 0.04 b	0.137 ± 0.038 b	0.48 ± 0.08 a	0.223 ± 0.015 a	0.0633 ± 0.0058 c	0.180 ± 0.040 a
*F* (cultivar)	18.71***	2.95*	1.88 ns	1.18 ns	11.03***	0.53 ns
Berry pulp—2024						
Concord	3.55 ± 0.34 ab	1.010 ± 0.080 b	2.87 ± 0.34 a	2.230 ± 0.140 a	0.257 ± 0.040 ab	0.390 ± 0.131 ab
Çeliksu	1.14 ± 0.14 e	0.253 ± 0.015 f	1.26 ± 0.26 c	0.403 ± 0.116 e	0.123 ± 0.015 cd	0.317 ± 0.015 ab
Niagara	2.36 ± 0.57 c	0.903 ± 0.074 bc	2.28 ± 0.18 ab	0.680 ± 0.070 cd	0.220 ± 0.010 bc	0.183 ± 0.103 b
Rizellim	3.70 ± 0.31 a	0.600 ± 0.060 d	2.90 ± 0.31 a	1.020 ± 0.110 bc	0.467 ± 0.071 a	0.543 ± 0.127 a
Rizessi	2.37 ± 0.05 bc	1.500 ± 0.230 a	3.12 ± 0.55 a	1.230 ± 0.160 b	0.420 ± 0.072 a	0.463 ± 0.143 ab
Rizpem	2.24 ± 0.10 c	0.330 ± 0.026 ef	1.60 ± 0.27 bc	0.627 ± 0.049 cde	0.210 ± 0.070 bcd	0.250 ± 0.135 ab
Steuben	1.37 ± 0.13 de	0.360 ± 0.046 e	1.38 ± 0.10 c	0.490 ± 0.010 de	0.113 ± 0.012 d	0.500 ± 0.056 ab
Ülkemiz	2.01 ± 0.55 cd	0.733 ± 0.127 cd	2.42 ± 0.29 a	1.340 ± 0.430 b	0.167 ± 0.074 bcd	n.d.
*F* (cultivar)	19.64***	77.63***	19.47***	31.88***	16.61***	6.74**
Berry pulp—2025						
Concord	0.99 ± 0.14 b	0.257 ± 0.051 b	1.32 ± 0.29 cd	0.383 ± 0.091 d	0.107 ± 0.012 c	0.337 ± 0.049 ab
Çeliksu	3.92 ± 0.20 a	0.417 ± 0.071 ab	2.94 ± 0.37 ab	0.683 ± 0.055 bc	0.223 ± 0.021 b	0.217 ± 0.032 ab
Niagara	4.67 ± 0.19 a	0.670 ± 0.100 a	3.00 ± 0.18 ab	1.170 ± 0.170 a	0.160 ± 0.030 bc	0.260 ± 0.026 ab
Rizellim	1.33 ± 0.20 b	0.280 ± 0.070 b	1.25 ± 0.22 d	0.397 ± 0.074 d	0.110 ± 0.017 c	0.313 ± 0.101 ab
Rizessi	4.68 ± 0.32 a	0.550 ± 0.123 a	2.29 ± 0.67 bc	1.090 ± 0.280 ab	0.153 ± 0.055 bc	0.180 ± 0.062 b
Rizpem	1.30 ± 0.19 b	0.273 ± 0.035 b	1.48 ± 0.14 cd	0.500 ± 0.026 cd	0.107 ± 0.035 c	0.367 ± 0.131 a
Steuben	4.55 ± 0.42 a	0.657 ± 0.220 a	4.37 ± 1.02 a	1.020 ± 0.220 ab	0.433 ± 0.075 a	0.360 ± 0.046 a
Ülkemiz	1.12 ± 0.10 b	0.230 ± 0.052 b	1.01 ± 0.21 d	0.417 ± 0.057 cd	0.107 ± 0.015 c	0.347 ± 0.042 a
*F* (cultivar)	134.02***	10.58***	19.75***	19.53***	14.40***	3.33*
Berry juice—2024						
Concord	4.81 ± 0.20 a	1.220 ± 0.120 a	3.65 ± 0.09 a	3.200 ± 0.240 a	0.383 ± 0.040 ab	0.600 ± 0.131 abc
Çeliksu	1.51 ± 0.27 c	0.287 ± 0.038 c	1.35 ± 0.02 d	0.500 ± 0.095 d	0.153 ± 0.057 c	0.507 ± 0.129 bc
Niagara	3.03 ± 0.55 b	0.923 ± 0.234 ab	3.17 ± 0.25 a	0.760 ± 0.157 cd	0.260 ± 0.066 bc	0.337 ± 0.025 c
Rizellim	4.19 ± 0.22 a	1.610 ± 0.900 a	3.64 ± 0.26 a	2.270 ± 0.680 ab	0.413 ± 0.199 ab	1.270 ± 0.790 a
Rizessi	4.62 ± 0.41 a	1.950 ± 0.110 a	3.82 ± 0.14 a	1.550 ± 0.070 b	0.630 ± 0.061 a	0.813 ± 0.055 ab
Rizpem	3.09 ± 0.10 b	0.460 ± 0.075 bc	2.41 ± 0.31 b	0.877 ± 0.068 c	0.267 ± 0.040 bc	0.373 ± 0.047 c
Steuben	1.94 ± 0.10 c	0.470 ± 0.085 bc	1.96 ± 0.17 c	0.587 ± 0.072 cd	0.137 ± 0.015 c	0.563 ± 0.065 abc
Ülkemiz	2.74 ± 0.25 b	0.920 ± 0.089 ab	2.56 ± 0.07 b	1.74 0 ± 0.110 b	0.193 ± 0.029 bc	n.d.
*F* (cultivar)	44.83***	13.18***	73.69***	45.95***	11.60***	6.99**
Berry juice—2025						
Concord	1.52 ± 0.26 c	0.297 ± 0.074 d	1.49 ± 0.25 b	0.537 ± 0.105 c	0.140 ± 0.036 c	0.433 ± 0.087 ab
Çeliksu	4.67 ± 0.42 a	0.470 ± 0.090 bcd	3.96 ± 0.85 a	0.860 ± 0.161 bc	0.327 ± 0.085 ab	0.277 ± 0.050 bc
Niagara	4.65 ± 0.31 a	0.903 ± 0.165 a	3.97 ± 1.10 a	1.660 ± 0.430 a	0.217 ± 0.084 abc	0.350 ± 0.177 ab
Rizellim	2.16 ± 0.12 b	0.467 ± 0.032 bcd	1.87 ± 0.08 b	0.600 ± 0.010 c	0.223 ± 0.035 abc	0.623 ± 0.040 a
Rizessi	4.97 ± 0.76 a	0.530 ± 0.053 abc	2.19 ± 0.20 b	1.090 ± 0.050 ab	0.137 ± 0.023 c	0.157 ± 0.015 c
Rizpem	1.97 ± 0.34 bc	0.500 ± 0.121 bcd	2.55 ± 0.16 ab	0.820 ± 0.075 bc	0.193 ± 0.064 bc	0.397 ± 0.025 ab
Steuben	4.55 ± 0.38 a	0.660 ± 0.144 ab	4.35 ± 0.87 a	1.150 ± 0.180 ab	0.487 ± 0.170 a	0.393 ± 0.093 ab
Ülkemiz	1.62 ± 0.14 bc	0.343 ± 0.095 cd	1.54 ± 0.48 b	0.567 ± 0.116 c	0.140 ± 0.000 bc	0.490 ± 0.079 ab
*F* (cultivar)	74.69***	8.93***	14.38***	16.43***	8.10***	8.52***

*Note:* Values are means ± standard deviation of three field replicates (*n* = 3) unless indicated otherwise; n.d., not determined. Within each fraction × season block, means sharing a letter do not differ (Tukey's HSD, α = 0.05, on log‐transformed data). *F* values test the simple effect of cultivar within each season (log(*y*) ~ Block + Cultivar), with Benjamini–Hochberg false‐discovery‐rate correction across all 104 fraction × element × season tests; ***q < 0.001, **q < 0.01, *q < 0.05, ns, not significant. Because the year × cultivar interaction was significant for 49 of 52 fraction × element combinations, cultivar means are reported separately by season rather than pooled across years. Absolute concentrations are provisional (Section 5) and should not be compared directly with values from other studies.

**FIGURE 3:**
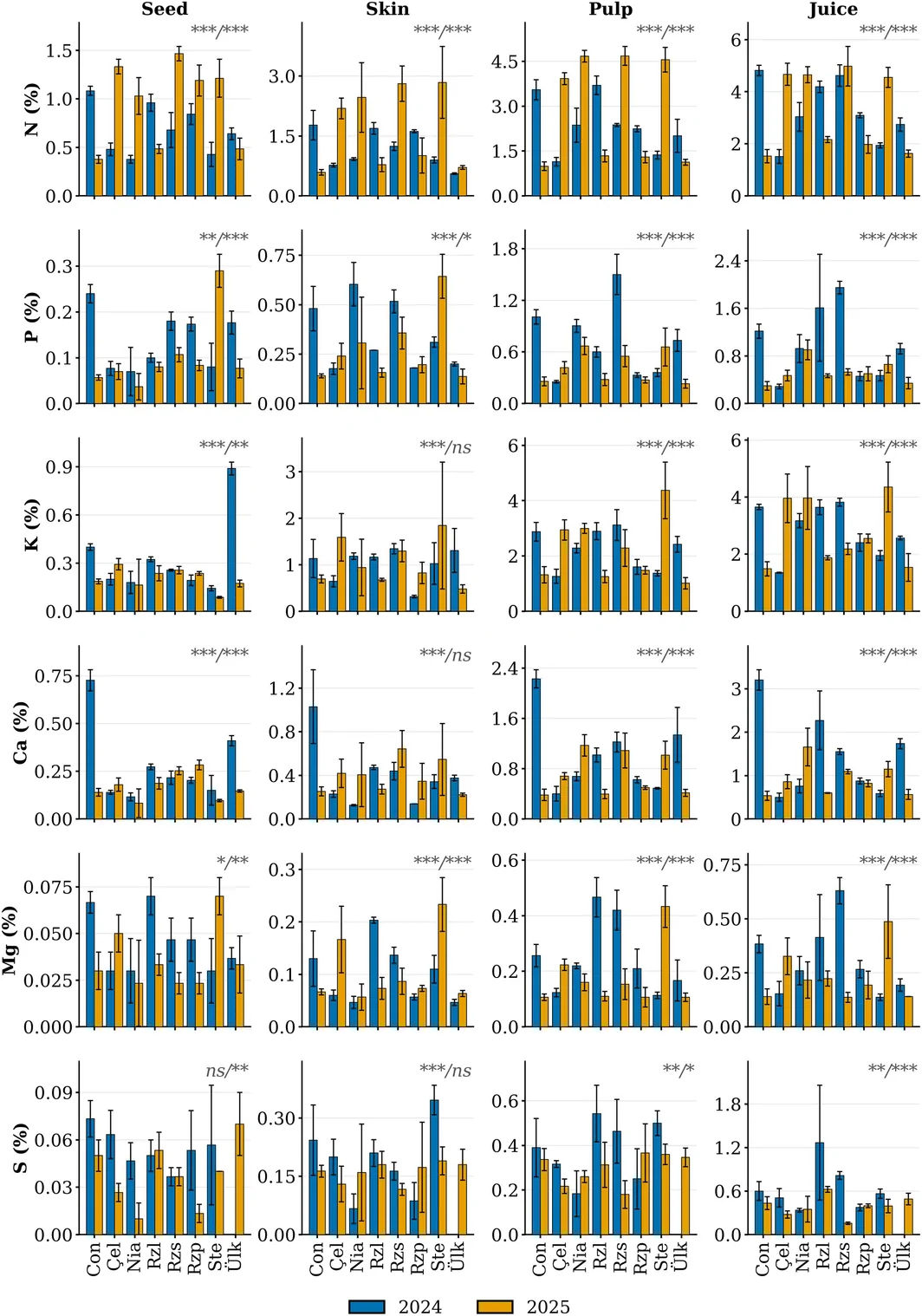
Macronutrient concentrations (% of dry weight) in four berry fractions of eight *Vitis labrusca* cultivars in the 2024 (blue) and 2025 (orange) growing seasons. Bars are means of three field replicates and error bars are standard deviations. Cultivars are abbreviated as Con, Concord; Çel, Çeliksu; Nia, Niagara; Rzl, Rizellim; Rzs, Rizessi; Rzp, Rizpem; Ste, Steuben; Ülk, Ülkemiz. The pair of symbols in the upper right of each panel gives the significance of the simple effect of cultivar in 2024 and 2025 respectively (***q < 0.001, **q < 0.01, *q < 0.05, ns, not significant, after Benjamini–Hochberg correction). Cultivar mean separation letters are given in Table 1 and are not repeated here. Absolute concentrations are provisional (Section 5), and no comparison is made between fractions.

In the skin fraction, nitrogen was highest in Concord in 2024 (1.77%) but in Steuben in 2025 (2.84%), Concord falling to the lowest value of the panel in the second season (0.59%). Phosphorus was highest in Niagara in 2024 (0.603%) and in Steuben in 2025 (0.643%), while magnesium was highest in Rizellim in 2024 (0.203%) and in Steuben in 2025 (0.233%), Niagara recording the lowest value in both seasons. Potassium, calcium, and sulfur differed among cultivars in 2024 but not in 2025 (*q* > 0.05), the loss of significance in the second season reflecting the markedly greater within‐cultivar variability recorded in the skin in 2025. In the pulp fraction, all six macronutrients differed among cultivars in both seasons. Nitrogen was highest in Rizellim in 2024 (3.70%) and in Rizessi in 2025 (4.68%), while Concord fell from 3.55% to 0.99%, the lowest value of the 2025 panel. Phosphorus was highest in Rizessi in 2024 (1.500%) and in Niagara in 2025 (0.670%), and calcium was highest in Concord in 2024 (2.230%) but in Niagara in 2025 (1.170%), Concord declining to the lowest value of the panel. Potassium was highest in Rizessi in 2024 (3.120%) and in Steuben in 2025 (4.373%), and magnesium likewise shifted from Rizellim (0.467%) to Steuben (0.433%) between seasons. In the juice fraction, the same pattern of ranking reversal was evident. Nitrogen was highest in Concord in 2024 (4.81%) and lowest in Concord in 2025 (1.52%), while Rizessi occupied the highest position in 2025 (4.97%). Potassium was highest in Rizessi in 2024 (3.820%) and in Steuben in 2025 (4.353%); calcium was highest in Concord in 2024 (3.200%) and in Niagara in 2025 (1.660%); and magnesium was highest in Rizessi in 2024 (0.630%) but lowest in the same cultivar in 2025 (0.137%), Steuben taking the highest position (0.487%). Sulfur was the only macronutrient for which the same cultivar, Rizellim, recorded the highest concentration in both seasons (1.270% and 0.623%).

### 3.2 Micronutrient Concentrations Within Berry Fractions

Micronutrient concentrations are presented in Table 2 and illustrated in Figure 4. Cultivar effects were significant in 49 of the 56 fraction × element × season combinations examined. In the seed fraction, iron was highest in Çeliksu in 2024 (18.29 mg kg^−1^) and in Rizellim in 2025 (20.71 mg kg^−1^), while zinc was highest in Çeliksu in both seasons, rising from 2.72 to 8.10 mg kg^−1^. Manganese differed among cultivars only in 2024, when Ülkemiz recorded the highest concentration (28.80 mg kg^−1^) and Steuben the lowest (4.47 mg kg^−1^); by 2025 the differences among cultivars were no longer significant (*q* > 0.05). Sodium was highest in Niagara in 2024 (97.56 mg kg^−1^) and in Concord in 2025 (153.81 mg kg^−1^), Steuben falling to 4.91 mg kg^−1^ in the second season. Chloride did not differ among cultivars in 2024 but did so in 2025, when Niagara recorded the highest value (1.69 mg kg^−1^). In the skin fraction, Steuben recorded the highest iron (77.55 mg kg^−1^), zinc (13.81 mg kg^−1^), and boron (3.32 mg kg^−1^) concentrations of the 2024 panel, and retained the highest zinc concentration in 2025 (71.40 mg kg^−1^). Manganese was highest in Ülkemiz in 2024 (27.80 mg kg^−1^) but did not differ significantly among cultivars in 2025, and the same loss of significance was observed for iron and boron. Sodium was highest in Rizessi in 2024 (310.28 mg kg^−1^) and in Concord in 2025 (521.42 mg kg^−1^).

**TABLE 2:** Micronutrient concentrations (mg kg^−1^ of dry weight) in four berry fractions of eight *Vitis labrusca* cultivars in the 2024 and 2025 growing seasons.

Cultivar	Mn	Fe	Zn	B	Cu	Na	Cl
Berry seed—2024							
Concord	15.3 ± 0.8 ab	6.06 ± 0.71 b	0.77 ± 0.06 bc	0.69 ± 0.15 bc	0.02 ± 0.00 c	28.4 ± 5.4 c	0.120 ± 0.017 a
Çeliksu	4.62 ± 1.22 c	18.3 ± 4.8 a	2.72 ± 0.50 a	1.09 ± 0.45 abc	21.10 ± 2.7 a	69.9 ± 20.6 ab	0.210 ± 0.020 a
Niagara	20.2 ± 6.8 ab	13.3 ± 5.6 a	0.92 ± 0.51 bc	0.63 ± 0.09 bc	9.39 ± 8.15 ab	97.6 ± 12.6 a	0.213 ± 0.084 a
Rizellim	20.6 ± 0.7 ab	4.42 ± 0.76 b	0.28 ± 0.07 c	1.97 ± 0.10 a	0.36 ± 0.04 bc	62.4 ± 4.7 ab	0.157 ± 0.012 a
Rizessi	13.4 ± 3.4 b	5.77 ± 0.22 b	0.25 ± 0.04 c	1.35 ± 0.28 ab	0.56 ± 0.01 b	85.1 ± 4.9 ab	0.123 ± 0.154 a
Rizpem	12.3 ± 1.7 b	5.74 ± 0.46 b	0.28 ± 0.09 c	1.61 ± 0.70 ab	0.27 ± 0.03 bc	57.7 ± 6.3 b	0.197 ± 0.015 a
Steuben	4.47 ± 1.75 c	17.7 ± 6.9 a	2.69 ± 1.86 ab	1.26 ± 0.80 abc	23.1 ± 8.1 a	91.9 ± 16.9 ab	0.190 ± 0.026 a
Ülkemiz	28.8 ± 0.6 a	5.31 ± 0.61 b	n.d.	0.45 ± 0.15 c	0.02 ± 0.00 c	n.d.	0.143 ± 0.025 a
*F* (cultivar)	27.33***	13.87***	15.14***	6.02**	15.85***	24.09***	1.62 ns
Berry seed—2025							
Concord	4.38 ± 0.40 a	17.3 ± 1.6 ab	2.30 ± 0.71 ab	0.673 ± 0.116 a	16.7 ± 1.5 a	154.0 ± 37.1 a	0.23 ± 0.06 bc
Çeliksu	7.07 ± 0.47 a	15.9 ± 0.7 ab	8.10 ± 2.27 a	0.983 ± 0.098 a	3.21 ± 1.96 b	53.8 ± 8.6 cd	1.33 ± 0.23 a
Niagara	3.50 ± 3.74 a	10.3 ± 6.5 ab	3.31 ± 0.77 ab	0.670 ± 0.849 a	2.36 ± 1.16 b	40.1 ± 6.0 d	1.69 ± 0.35 a
Rizellim	5.23 ± 0.90 a	20.7 ± 3.5 a	2.47 ± 0.34 ab	0.973 ± 0.121 a	19.6 ± 1.8 a	98.7 ± 9.3 ab	0.04 ± 0.00 d
Rizessi	4.41 ± 0.69 a	9.75 ± 2.26 ab	1.19 ± 0.38 b	0.803 ± 0.282 a	0.06 ± 0.01 c	31.8 ± 4.0 d	1.32 ± 1.27 ab
Rizpem	3.11 ± 1.08 a	7.45 ± 1.84 b	0.407 ± 0.592 c	0.320 ± 0.115 a	0.04 ± 0.00 c	33.5 ± 7.1 d	0.54 ± 0.09 ab
Steuben	4.21 ± 2.01 a	10.3 ± 4.3 ab	2.87 ± 0.88 ab	0.843 ± 0.447 a	0.01 ± 0.00 d	4.91 ± 0.81 e	1.37 ± 0.85 ab
Ülkemiz	5.19 ± 1.59 a	20.6 ± 6.3 a	2.36 ± 0.49 ab	1.24 ± 0.65 a	26.2 ± 12.8 a	79.4 ± 28.7 bc	0.09 ± 0.11 cd
*F* (cultivar)	2.50 ns	9.40***	14.13***	2.69 ns	554.09***	71.53***	30.55***
Berry skin—2024							
Concord	23.7 ± 3.5 ab	12.8 ± 5.9 b	2.52 ± 0.32 c	0.88 ± 0.27 c	0.04 ± 0.05 f	29.5 ± 3.8 c	0.280 ± 0.017 a
Çeliksu	10.6 ± 7.4 c	43.5 ± 11.7 a	8.27 ± 2.30 b	1.32 ± 0.65 bc	44.10 ± 3.30 b	210 ± 43 a	0.387 ± 0.116 a
Niagara	19.7 ± 2.9 ab	15.6 ± 1.3 b	0.97 ± 0.01 d	0.56 ± 0.08 c	0.06 ± 0.01 f	247 ± 48 a	0.510 ± 0.078 a
Rizellim	18.8 ± 1.7 abc	12.7 ± 2.3 b	1.05 ± 0.08 d	2.32 ± 1.04 ab	0.70 ± 0.03 d	63.2 ± 12.0 b	0.310 ± 0.010 a
Rizessi	21.7 ± 2.3 ab	11.5 ± 3.6 b	0.86 ± 0.06 d	2.48 ± 0.47 ab	1.26 ± 0.07 c	310 ± 62 a	0.370 ± 0.255 a
Rizpem	17.1 ± 1.5 abc	18.2 ± 1.6 b	1.13 ± 0.08 d	1.10 ± 0.27 bc	0.30 ± 0.03 e	29.7 ± 0.9 c	0.340 ± 0.053 a
Steuben	13.1 ± 2.7 bc	77.5 ± 16.3 a	13.80 ± 2.70 a	3.32 ± 0.79 a	80.20 ± 15.30 a	252 ± 96 a	0.267 ± 0.015 a
Ülkemiz	27.8 ± 0.4 a	18.2 ± 4.0 b	n.d.	0.20 ± 0.05 d	0.02 ± 0.00 g	n.d.	0.173 ± 0.025 a
*F* (cultivar)	5.11**	20.23***	210.57***	22.45***	2778.54***	83.38***	1.78 ns
Berry skin—2025							
Concord	7.16 ± 0.28 a	42.5 ± 1.7 a	6.47 ± 1.00 ab	1.54 ± 0.24 a	41.6 ± 2.7 a	521 ± 72 a	0.38 ± 0.04 bc
Çeliksu	23.4 ± 9.9 a	82.0 ± 36.5 a	55.3 ± 27.1 a	4.06 ± 2.16 a	18.6 ± 12.0 a	222 ± 35 ab	2.37 ± 0.32 ab
Niagara	8.18 ± 4.95 a	26.7 ± 8.0 a	8.36 ± 2.10 ab	0.97 ± 0.60 a	2.05 ± 3.23 a	150 ± 12 ab	3.05 ± 0.49 a
Rizellim	8.05 ± 0.19 a	47.8 ± 1.1 a	6.68 ± 0.95 ab	1.89 ± 0.44 a	44.6 ± 16.1 a	453 ± 104 ab	0.09 ± 0.01 c
Rizessi	8.66 ± 0.48 a	27.4 ± 2.2 a	4.25 ± 0.33 ab	1.33 ± 0.35 a	0.16 ± 0.02 a	128 ± 29 ab	3.65 ± 1.94 a
Rizpem	7.54 ± 4.03 a	42.1 ± 27.5 a	7.36 ± 7.19 b	1.48 ± 1.29 a	35.8 ± 31.1 a	210 ± 60 ab	0.46 ± 0.68 c
Steuben	24.5 ± 18.6 a	80.8 ± 58.3 a	71.4 ± 56.5 ab	3.39 ± 2.53 a	46.1 ± 40.0 a	154 ± 119 b	2.54 ± 0.66 a
Ülkemiz	7.52 ± 0.66 a	44.7 ± 3.9 a	8.92 ± 1.60 ab	1.71 ± 0.35 a	51.8 ± 2.5 a	401 ± 28 ab	0.37 ± 0.03 bc
*F* (cultivar)	2.16 ns	1.78 ns	3.99*	1.72 ns	3.22*	4.55**	15.24***
Berry pulp—2024							
Concord	48.8 ± 3.4 a	27.8 ± 5.7 c	6.32 ± 1.07 b	1.62 ± 0.40 ab	0.143 ± 0.015 e	91.3 ± 0.9 b	0.360 ± 0.066 c
Çeliksu	11.3 ± 1.4 f	67.0 ± 8.4 b	14.8 ± 4.9 a	2.65 ± 0.59 ab	80.6 ± 20.1 a	525.0 ± 73.0 a	0.483 ± 0.045 c
Niagara	21.0 ± 4.0 de	28.9 ± 2.6 c	1.71 ± 0.15 d	3.07 ± 0.47 ab	0.23 ± 0.00 d	587.0 ± 39.0 a	0.837 ± 0.129 b
Rizellim	39.9 ± 2.8 ab	24.1 ± 2.4 c	2.59 ± 0.48 cd	6.74 ± 4.59 a	0.17 ± 0.06 de	49.6 ± 15.0 c	0.490 ± 0.035 c
Rizessi	33.7 ± 2.5 bc	25.5 ± 2.7 c	3.07 ± 0.77 c	2.75 ± 0.85 ab	2.99 ± 0.28 b	129.0 ± 11.0 b	1.380 ± 0.170 a
Rizpem	27.6 ± 3.5 cd	27.9 ± 0.9 c	1.93 ± 0.14 cd	4.58 ± 2.34 ab	1.24 ± 0.16 c	40.8 ± 2.1 c	0.460 ± 0.036 c
Steuben	17.6 ± 2.2 e	104 ± 13 a	19.7 ± 2.8 a	4.22 ± 1.34 ab	112.00 ± 2.00 a	488.0 ± 9.0 a	0.500 ± 0.082 c
Ülkemiz	30.6 ± 4.5 bc	25.8 ± 1.1 c	n.d.	1.19 ± 0.09 b	0.06 ± 0.05 f	n.d.	0.450 ± 0.020 c
*F* (cultivar)	39.66***	57.09***	75.30***	3.42*	1234.24***	193.67***	34.17***
Berry pulp—2025							
Concord	10.8 ± 2.5 bc	63.7 ± 15.0 cde	12.8 ± 2.9 cd	2.95 ± 0.72 bcd	77.3 ± 7.7 a	873 ± 25 a	0.61 ± 0.07 b
Çeliksu	40.5 ± 2.7 a	116.0 ± 37.0 ab	64.0 ± 37.7 b	5.70 ± 3.61 ab	25.0 ± 19.4 b	425 ± 45 bc	3.62 ± 0.74 a
Niagara	16.3 ± 1.0 b	47.8 ± 0.4 de	10.5 ± 2.3 cd	2.05 ± 0.05 cd	0.29 ± 0.09 c	282 ± 11 c	4.65 ± 1.23 a
Rizellim	14.3 ± 4.3 bc	84.7 ± 25.5 bcd	14.7 ± 2.4 cd	2.78 ± 0.48 bcd	90.8 ± 9.9 a	717 ± 17 ab	0.12 ± 0.03 c
Rizessi	11.7 ± 3.2 bc	38.5 ± 7.5 e	7.5 ± 0.6 d	1.48 ± 0.32 d	0.40 ± 0.04 c	295 ± 38 c	2.54 ± 0.57 a
Rizpem	14.4 ± 0.9 bc	85.3 ± 5.1 abc	18.5 ± 6.3 c	3.30 ± 0.47 bc	75.5 ± 22.8 a	403 ± 79 c	0.13 ± 0.01 c
Steuben	51.8 ± 4.6 a	149.0 ± 8.0 a	157.0 ± 41.0 a	8.38 ± 1.55 a	67.0 ± 25.0 a	401 ± 28 c	7.75 ± 7.58 a
Ülkemiz	9.39 ± 2.28 c	55.6 ± 13.5 cde	15.6 ± 0.8 cd	2.40 ± 0.72 bcd	78.8 ± 11.6 a	785 ± 20 a	0.67 ± 0.08 b
*F* (cultivar)	35.22***	19.65***	47.39***	11.93***	203.47***	16.30***	58.44***
Berry juice—2024							
Concord	67.8 ± 3.3 a	37.6 ± 4.4 b	8.00 ± 0.76 bc	3.20 ± 1.32 ab	0.11 ± 0.02 f	63.6 ± 6.4 d	0.42 ± 0.01 b
Çeliksu	19.1 ± 2.5 c	107.0 ± 14.0 a	13.80 ± 0.70 ab	4.04 ± 1.56 a	80.7 ± 7.2 b	387.0 ± 98.0 b	0.75 ± 0.04 ab
Niagara	41.8 ± 11.3 b	36.9 ± 6.0 b	3.06 ± 0.72 de	4.52 ± 0.73 a	0.29 ± 0.02 e	789.0 ± 21.0 a	1.04 ± 0.60 ab
Rizellim	65.3 ± 14.1 a	30.4 ± 2.9 b	6.40 ± 3.15 cd	2.70 ± 0.54 ab	0.160 ± 0.02 f	79.5 ± 17.3 cd	0.65 ± 0.07 b
Rizessi	51.3 ± 5.4 ab	37.0 ± 4.0 b	4.23 ± 0.33 cde	3.86 ± 0.48 a	3.11 ± 0.43 c	117.0 ± 11.0 c	1.87 ± 0.69 a
Rizpem	33.2 ± 5.5 b	34.0 ± 1.8 b	2.45 ± 0.36 e	5.61 ± 1.39 a	1.50 ± 0.13 d	86.1 ± 11.0 cd	0.72 ± 0.10 b
Steuben	14.6 ± 1.1 c	82.3 ± 6.1 a	19.80 ± 5.20 a	3.09 ± 0.73 ab	140 ± 39 a	823.0 ± 95.0 a	0.76 ± 0.08 ab
Ülkemiz	37.6 ± 2.6 b	26.9 ± 6.5 b	n.d.	1.65 ± 0.20 b	0.12 ± 0.06 f	n.d.	0.54 ± 0.07 b
*F* (cultivar)	40.81***	44.70***	26.87***	5.74**	995.48***	187.68***	4.74**
Berry juice—2025							
Concord	15.9 ± 1.2 c	89.5 ± 6.8 abc	14.5 ± 1.4 de	4.19 ± 1.00 ab	90.8 ± 28.1 ab	1284 ± 417 a	0.95 ± 0.23 d
Çeliksu	43.8 ± 14.4 a	109.0 ± 8.0 ab	44.5 ± 0.9 b	5.42 ± 0.61 ab	18.0 ± 1.4 c	502 ± 38 bcd	6.38 ± 0.47 ab
Niagara	23.7 ± 1.5 abc	70.1 ± 6.5 bc	14.6 ± 1.1 de	3.42 ± 0.41 ab	0.4 ± 0.1 d	384 ± 34 cd	6.67 ± 3.69 ab
Rizellim	23.2 ± 2.1 abc	130.0 ± 12.0 a	26.8 ± 2.3 bc	5.20 ± 0.66 ab	153.0 ± 6.0 a	804 ± 120 ab	0.14 ± 0.02 e
Rizessi	16.9 ± 1.3 bc	54.9 ± 3.1 c	10.5 ± 0.3 e	2.00 ± 0.26 b	0.4 ± 0.0 d	290 ± 31 d	3.65 ± 1.41 bc
Rizpem	21.8 ± 4.7 abc	123.0 ± 26.0 ab	24.2 ± 1.7 bcd	4.19 ± 1.11 ab	82.1 ± 8.2 ab	870 ± 269 ab	0.19 ± 0.02 e
Steuben	41.5 ± 26.5 ab	121.0 ± 51.0 ab	142 ± 35.0 a	8.33 ± 7.53 a	55.5 ± 32.3 b	643 ± 30 bc	15.6 0 ± 9.50 a
Ülkemiz	16.8 ± 6.1 c	94.7 ± 34.4 abc	24.7 ± 12.0 cd	3.02 ± 1.20 ab	101.0 ± 29.0 ab	1325 ± 401 a	1.29 ± 0.49 cd
*F* (cultivar)	4.98**	5.48**	49.38***	2.97*	238.16***	15.17***	66.31***

*Note:* Values are means ± standard deviation of three field replicates (*n* = 3) unless indicated otherwise; n.d., not determined. Within each fraction × season block, means sharing a letter do not differ (Tukey's HSD, α = 0.05, on log‐transformed data). *F* values test the simple effect of cultivar within each season, with Benjamini–Hochberg correction as in Table 1. Copper is reported for completeness but is not interpreted; see Section 5. Absolute concentrations are provisional (Section 5).

**FIGURE 4:**
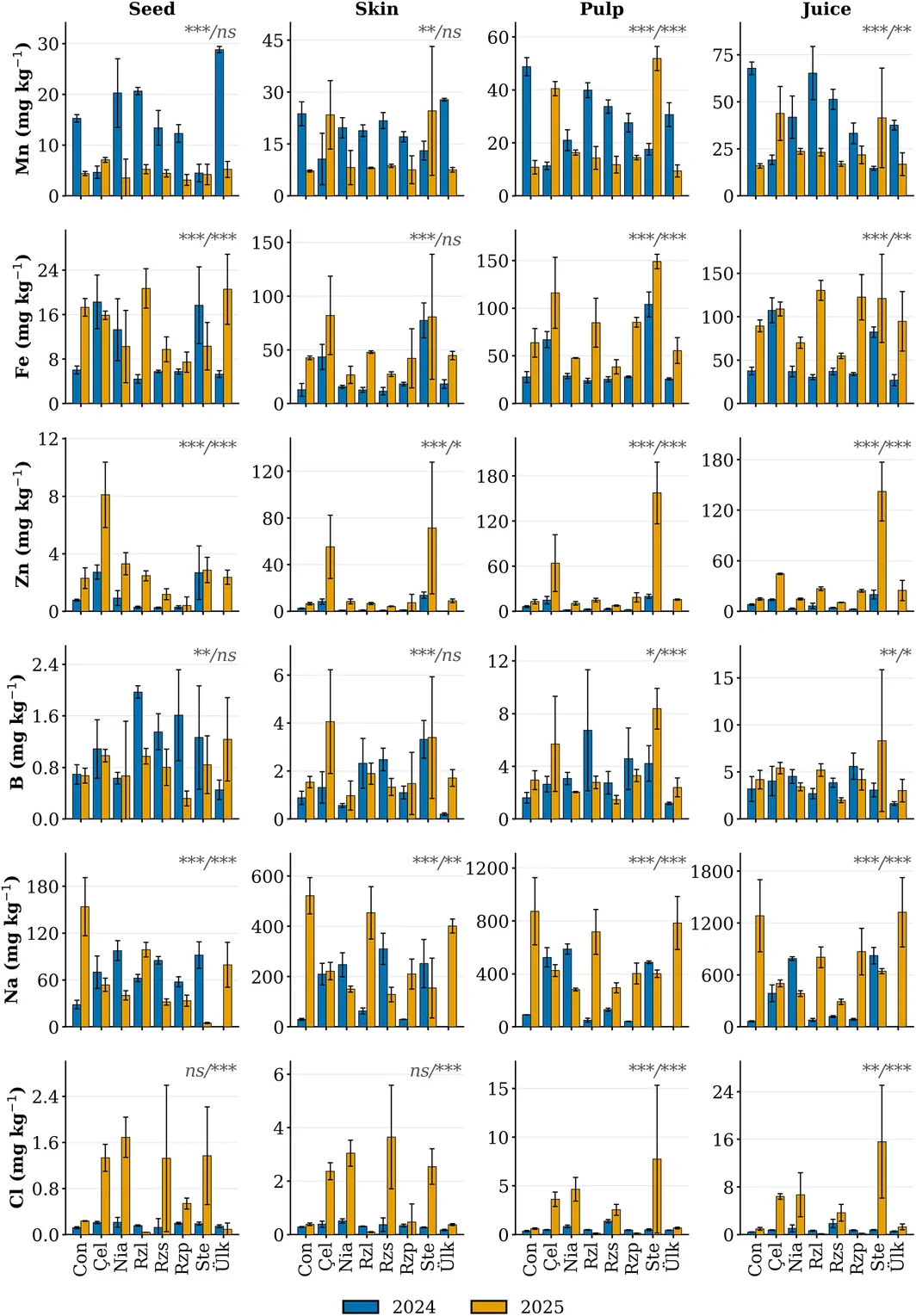
Micronutrient concentrations (mg kg^−1^ of dry weight) in four berry fractions of eight *Vitis labrusca* cultivars in the 2024 (blue) and 2025 (orange) growing seasons. Bars, error bars, cultivar abbreviations and significance symbols are as described for Figure 3. Copper is reported in Table 2 but is not shown here because it was not interpreted (Section 5). Cultivar mean separation letters are given in Table 2.

In the pulp fraction, Steuben recorded the highest iron concentration in both seasons (103.97 and 148.94 mg kg^−1^) and the highest zinc concentration in both seasons, the latter rising from 19.70 to 157.33 mg kg^−1^. Manganese was highest in Concord in 2024 (48.78 mg kg^−1^) and in Steuben in 2025 (51.84 mg kg^−1^). Boron shifted from Rizellim (6.74 mg kg^−1^) to Steuben (8.38 mg kg^−1^) between seasons, and sodium from Niagara (586.86 mg kg^−1^) to Concord (873.08 mg kg^−1^). Chloride was highest in Rizessi in 2024 (1.38 mg kg^−1^) and in Steuben in 2025 (7.75 mg kg^−1^). In the juice fraction, manganese was highest in Concord in 2024 (67.83 mg kg^−1^) and lowest in the same cultivar in 2025 (15.89 mg kg^−1^), Çeliksu taking the highest position in the second season (43.80 mg kg^−1^). Iron was highest in Çeliksu in 2024 (107.44 mg kg^−1^) and in Rizellim in 2025 (130.42 mg kg^−1^). Zinc was highest in Steuben in both seasons, rising from 19.80 to 142.12 mg kg^−1^, a value that exceeded the concentration of every other cultivar by at least fourfold. Sodium was highest in Steuben in 2024 (823.00 mg kg^−1^) and in Ülkemiz in 2025 (1325.02 mg kg^−1^); the latter value is a single‐season mean (*n* = 3), because the 2024 sodium values for this cultivar were lost to the data‐entry error documented in Table S1. Chloride was highest in Steuben in 2025 (15.60 mg kg^−1^). Copper is reported in Table 2 for completeness but is not interpreted here. Copper concentrations reached 153.44 mg kg^−1^ in the 2025 juice of Rizellim and differed among cultivar × fraction × season combinations by more than four orders of magnitude, despite the fact that no copper‐containing product was applied to the vineyard in either season. These values could not be reconciled with the management history of the site and were therefore treated as analytically unverified (Section 5).

### 3.3 Interannual Change in Mineral Composition

Interannual change is summarized in Figure 5, which presents the log_2_ ratio of the 2025 to the 2024 mean for every cultivar × fraction × element combination together with the corresponding false‐discovery‐rate‐adjusted significance. Of the 404 comparisons that could be evaluated, 208 were significant at *q* < 0.05, of which 110 represented increases and 98 decreases in 2025. Although the balance of increases and decreases was almost even overall, individual elements behaved with striking directional consistency. Every one of the 16 significant changes in iron and every one of the 20 significant changes in zinc was an increase in 2025, without a single significant decrease for either element in any cultivar or fraction. Manganese behaved in the opposite manner, 17 of its 19 significant changes being decreases, and phosphorus likewise declined in 13 of 14 significant comparisons. Calcium decreased in 13 of 18 and potassium in 11 of 16 significant comparisons, whereas chloride increased in 16 of 22 and sodium in 13 of 19. Sulfur changed significantly in only four comparisons, all of them decreases. Nitrogen was the element for which the direction of change was most evenly divided, with 11 increases and 10 decreases, reflecting the cultivar‐dependent reversals described in Sections 3.1 and 3.2; such variability is consistent with the marked sensitivity of berry nitrogen status to seasonal and vine‐management factors reported by Bell and Henschke (2005). The cultivars differed markedly in how extensively their composition changed between seasons. Concord and Rizellim showed significant interannual change across the greatest number of elements × fraction combinations and in all four fractions, whereas Niagara and Rizpem showed the fewest. Concord was the most variable cultivar in the seed and skin, and Rizellim in the pulp and juice. This pattern is quantified in Section 3.4.

**FIGURE 5:**
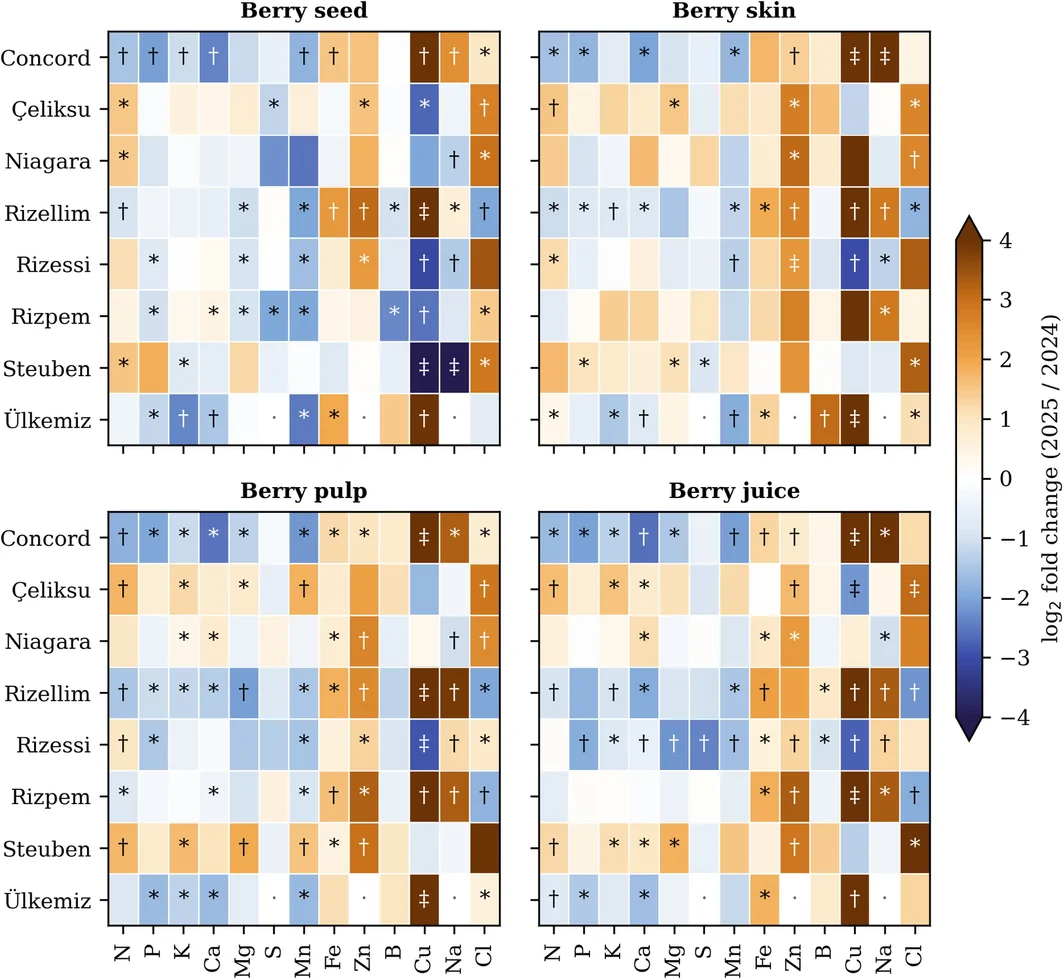
Interannual change in mineral concentration between the 2024 and 2025 seasons for every cultivar × element × fraction combination, expressed as the log_2_ ratio of the 2025 to the 2024 mean. Orange denotes a higher concentration in 2025 and blue a higher concentration in 2024. Symbols denote significance after Benjamini–Hochberg correction across all 404 comparisons (*q < 0.05, ^†^ *q* < 0.01, ^‡^ *q* < 0.001); a mid‐point dot indicates a combination that could not be evaluated because of the missing values documented in Table S1. The color scale is capped at ±4, and 21 of the 404 cells exceed this range and are shown in the terminal color.

### 3.4 Sources of Variation and Cultivar Stability

The partitioning of the total variance of each fraction × element combination into year, cultivar, year × cultivar and residual components is presented in Figure 2A and in full in Table S3. The year × cultivar interaction was significant for 49 of the 52 combinations and, averaged across all combinations, accounted for the largest single share of the total variance (39.4%), exceeding the shares attributable to cultivar (31.5%) and to year (13.6%); the residual accounted for the remaining 15.5%. The three combinations for which the interaction was not significant were sulfur, iron, and zinc, all in the skin fraction. The interaction component was not distributed evenly among fractions. It accounted for 38% of the total variance in the seed, 31% in the skin, 43% in the pulp, and 44% in the juice, so that the two fleshy fractions, which constitute the edible portion of the berry, were also those in which cultivar behavior was most strongly season‐dependent. The largest individual interaction components were recorded for manganese in the pulp (74%), nitrogen in the pulp (72%), potassium in the juice (72%), and copper in the seed (71%). By contrast, the cultivar component exceeded half of the total variance only for zinc in the seed (60%), zinc in the pulp (55%), chloride in the pulp (54%), iron in the pulp (54%), and zinc in the skin (51%), and the year component exceeded 40% only for iron in the juice (48%) and manganese in the seed (46%).

The consequence of a dominant interaction term is that cultivars cannot be ranked consistently across seasons, and this is directly visible in the reversals reported in Sections 3.1 and 3.2. It also means that the stability of a cultivar's composition between seasons is itself a property that varies among cultivars. Expressed as the median absolute log_2_ fold change across all 13 elements and all four fractions (Figure 2B), interannual instability ranged from 1.75‐fold in Niagara to 2.87‐fold in Concord, the full ranking being Niagara (1.75) < Rizpem (1.94) < Steuben (2.03) < Çeliksu (2.14) < Rizessi (2.21) < Ülkemiz (2.39) < Rizellim (2.77) < Concord (2.87). Resolving this measure by fraction revealed that stability is not a uniform property of a cultivar. Niagara and Rizpem, the two most stable cultivars overall, owed their stability principally to the pulp and juice fractions (1.50‐ and 1.91‐fold change in the pulp and 1.63‐ and 1.53‐fold in the juice respectively), while being comparatively variable in the seed and skin (2.43 and 2.00‐fold in the seed). Concord was the most variable cultivar in the seed (2.88‐fold) and skin (3.00‐fold), whereas Rizellim was the most variable in the pulp (2.79‐fold) and juice (3.45‐fold), the latter being the largest value recorded for any cultivar × fraction combination. Because these measures are ratios, they are unaffected by any constant scaling factor applied within a fraction and are therefore independent of the analytical limitations described in Section 5. Soluble solids concentration at harvest, tested as a covariate at the cultivar × year level, was not significantly associated with the concentration of any element in any fraction (0 of 52 tests significant; mean variance explained 4.0%, maximum 20.6%). The differences in harvest maturity among cultivars, which spanned 17.6–22.8 °Brix, therefore do not account for the mineral variation reported here.

### 3.5 Associations Among Mineral Elements

Partial correlations among elements, computed on residuals after cultivar, year and block effects had been removed, are presented in Figure 6. Of the 312 elemental pairs examined across the four fractions, 186 remained significantly correlated after false‐discovery‐rate correction, indicating that the associations among elements are not simply a consequence of the cultivars or seasons in which they were measured. The strongest positive associations differed among fractions but shared a common structure. In the pulp, phosphorus and potassium (*r* = +0.91), nitrogen and calcium (*r* = +0.90) and potassium and calcium (*r* = +0.88) were most strongly associated; in the juice, potassium and manganese (*r* = +0.89), phosphorus and potassium (*r* = +0.87) and potassium and calcium (*r* = +0.87); in the seed, copper and sodium (*r* = +0.81), phosphorus and magnesium (*r* = +0.81) and potassium and calcium (*r* = +0.80); and in the skin, copper and sodium (*r* = +0.78), nitrogen and chloride (*r* = +0.76) and potassium and manganese (*r* = +0.75). A consistent feature of all four fractions was that copper, sodium and iron were positively associated with one another and negatively associated with the group of elements comprising nitrogen, phosphorus, potassium, calcium, magnesium and manganese. The negative associations between nitrogen and copper (*r* = −0.80 in the seed, −0.90 in the pulp, −0.86 in the juice) and between nitrogen and sodium (*r* = −0.76 in the seed and skin, −0.87 in the pulp, −0.84 in the juice) were among the strongest recorded in the study. This opposition of two element groups was reproduced in every fraction and is considered further in Section 4.3.

**FIGURE 6:**
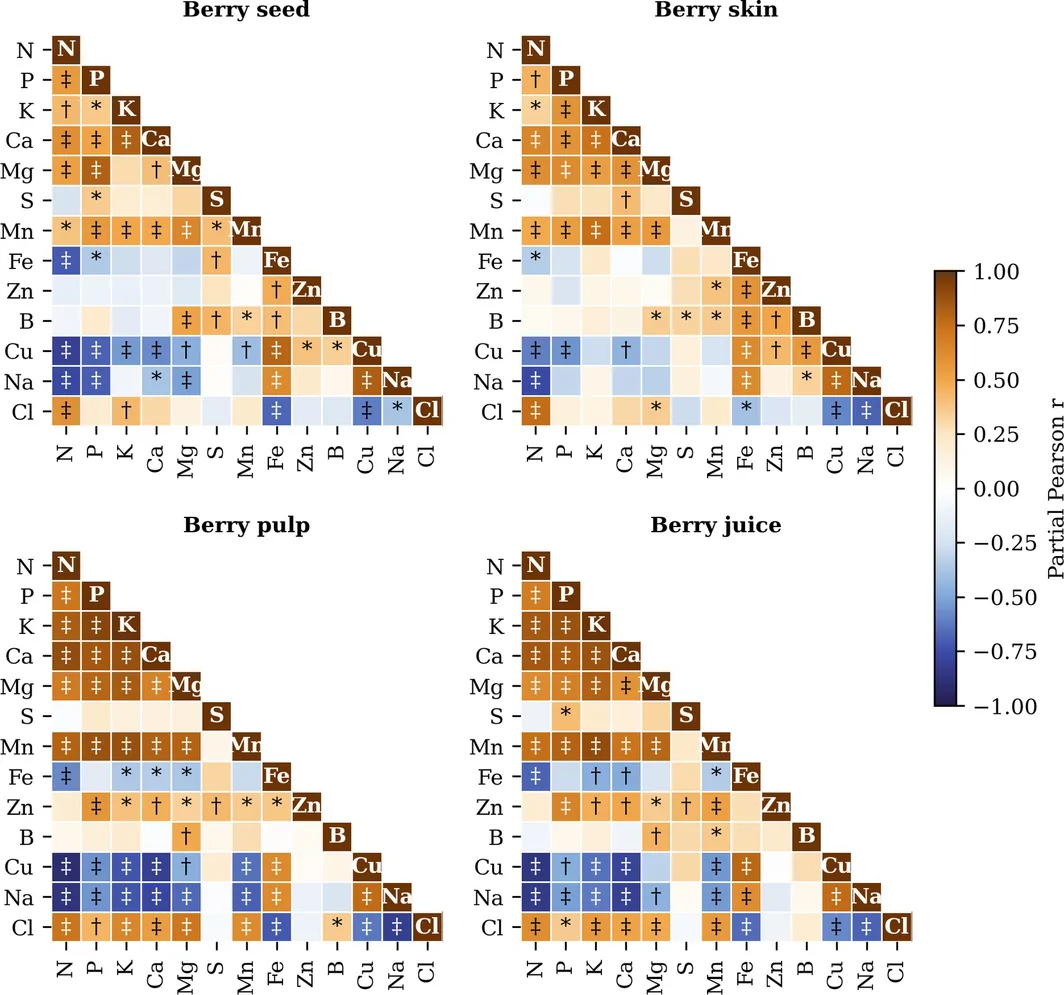
Partial correlations among mineral elements within each berry fraction, computed on residuals after the effects of cultivar, year and block had been removed. Symbols denote significance after Benjamini–Hochberg correction across all 312 pairwise tests (*q < 0.05, ^†^ *q* < 0.01, ^‡^ *q* < 0.001). Correlations computed in this way describe associations among elements that are not attributable to differences among cultivars or between seasons.

### 3.6 Multivariate Structure of Mineral Composition

Principal component analysis was performed on replicate‐level observations within each fraction (*n* = 44–45), and the resulting biplots are presented in Figure 7. The first two components accounted for 63.9% of the total variance in the seed (PC1 35.8%, PC2 28.1%), 63.3% in the skin (33.9%, 29.4%), 69.6% in the pulp (47.5%, 22.1%) and 67.6% in the juice (50.3%, 17.3%). The first component was dominated in every fraction by the macronutrient group, the largest loadings being phosphorus, calcium, magnesium and manganese in the seed, potassium, magnesium, calcium and boron in the skin, calcium, phosphorus, potassium and magnesium in the pulp, and calcium, phosphorus, manganese and copper in the juice. The loading vectors for copper, sodium and iron consistently opposed those of the macronutrient group along this axis, reproducing the structure observed in the partial correlations. Observations from the two seasons occupied clearly distinct regions of the biplot in every fraction, with the separation being most pronounced along PC1 in the pulp and juice. Cultivars were not separated consistently across seasons: several cultivars that were adjacent in 2024 were widely separated in 2025 and vice versa. This absence of a season‐independent cultivar arrangement is the multivariate expression of the dominant year × cultivar interaction quantified in Section 3.4, and it indicates that the mineral profile of a cultivar in this panel cannot be characterized from a single season.

**FIGURE 7:**
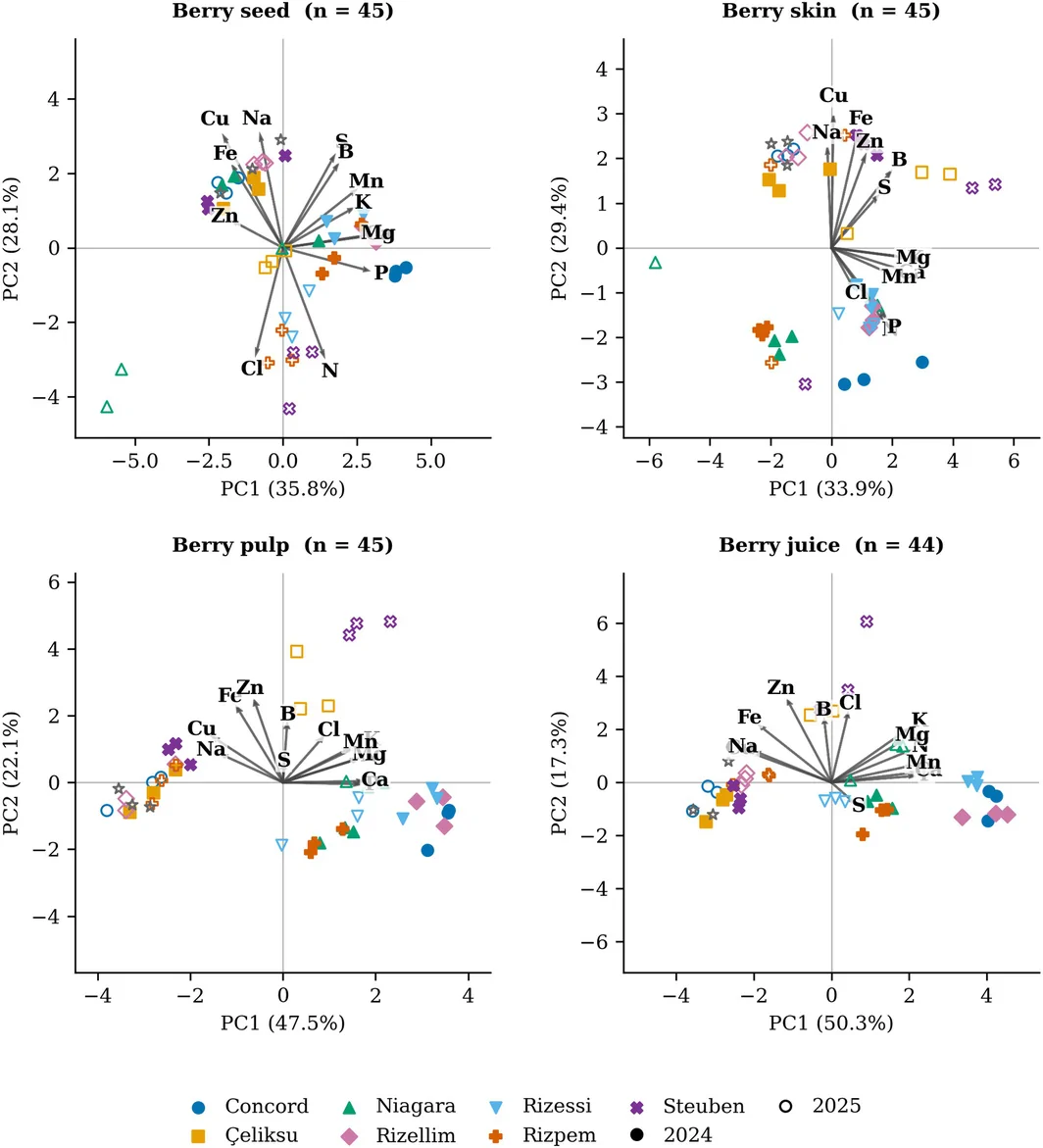
Principal component analysis of mineral composition within each berry fraction, performed on log‐transformed and standardized replicate‐level observations (*n* = 44–45 per fraction). Filled symbols denote the 2024 season and open symbols the 2025 season. Arrows are the loading vectors of the 13 elements, and the percentage of total variance accounted for by each component is given on the corresponding axis.

## 4 Discussion

### 4.1 Cultivar Variation in Macronutrient and Micronutrient Concentrations

The macronutrient profiles observed across berry fractions in the present study revealed pronounced inter‐cultivar variation that was both fraction‐specific and element‐dependent, and that was expressed differently in the two seasons. Potassium was among the most variable macronutrients in the seed fraction, with Ülkemiz recording the highest concentration in 2024, which is consistent with the well‐established role of potassium as the most abundant mineral cation in grape berry tissues. This is in general agreement with Rogiers et al. (2000, 2006), who reported potassium as the principal macronutrient accumulating predominantly in the pulp and skin of *V. vinifera* cv. Shiraz, and with Keskin et al. (2021), who found potassium to be the most abundant macroelement in the berries of Katıkara (*V. vinifera*) and Isabella (*V. labrusca*), at 1820.7 and 1495.9 mg/kg, respectively. Potassium's function in maintaining osmotic potential and driving phloem sap flow toward the berry sink offers a plausible physiological context for its abundance in the fleshy fractions, although the present design does not permit this mechanism to be tested directly. In grapevine, potassium is the most abundant cation of the berry; it is delivered to the fruit predominantly via the phloem, accumulates mainly in the skin and pulp during ripening, and contributes to osmotic adjustment, berry expansion and juice pH (Mpelasoka et al. 2003; Keller 2015). Phosphorus differentiated cultivars consistently within the skin, pulp and juice fractions, a pattern not previously emphasized in the grape mineral nutrition literature for *V. labrusca* cultivars. Al Juhaimi et al. (2017) reported phosphorus contents in grape seeds ranging from 2277.65 to 3232.42 mg/kg across 11 cultivars; the inter‐cultivar spread observed in the present panel was notably wider, which may reflect the broader genetic diversity of the *V. labrusca* material examined here, although a direct comparison of absolute values is not appropriate for the reasons set out in Section 5.

Micronutrient distributions revealed equally strong cultivar‐dependent patterns, of which the position of Steuben was the most consistent. Steuben recorded the highest zinc concentration in the skin, pulp, and juice in both seasons, and the highest iron concentration in the pulp in both seasons, making it the only cultivar in the panel whose ranking for these elements was stable across years. Székelyhidi et al. (2024) recorded iron concentrations ranging from 23.7 to 59.5 mg/kg in grape seeds across eight cultivars; the seed values observed here were lower than this range, whereas the concentrations recorded in the fleshy fractions were considerably higher. Whether this reflects a genuine characteristic of *V. labrusca* or the analytical uncertainty discussed in Section 5 cannot be resolved from the present data. Kurt et al. (2017), working on the hybrid cultivar ‘Isabel’, likewise reported that mineral concentrations in a *V. labrusca* ‐derived genotype shift substantially over the course of berry maturation, which underlines the sensitivity of these genotypes to the developmental and seasonal context in which they are sampled. Niagara and Rizessi were most frequently associated with the lowest micronutrient concentrations, a pattern that was reasonably consistent between seasons and that may indicate a lower mineral loading capacity in these cultivars, although a single site over two seasons cannot separate genotype from site‐specific effects.

### 4.2 The Dominance of Genotype × Year Interaction and Compositional Stability

The most substantial finding of the present study is that the year × cultivar interaction, rather than either factor alone, was the dominant source of variation in the mineral composition of these berries. Averaged across all fraction × element combinations, the interaction accounted for 39% of the total variance against 32% for cultivar and 14% for year, and it was significant in 49 of 52 combinations. To our knowledge this partitioning has not previously been reported for berry fractions of any *Vitis* species, and its implications are considerable for how mineral profiling studies in grapevine are designed and interpreted. The practical consequence is that cultivar rankings established in one season do not carry over to the next. This was observed repeatedly and across both nutrient groups: Concord recorded the highest seed nitrogen concentration of the panel in 2024 and the lowest in 2025; the highest juice calcium concentration in 2024 and the lowest in 2025; and the highest pulp manganese concentration in 2024, while Steuben occupied that position in 2025. Such reversals cannot be accommodated within an additive model of cultivar and year effects, and they mean that a single‐season characterization of a cultivar's mineral profile is liable to be misleading. This provides a quantitative basis for the general observation of Colak and Bengu (2024) that year‐to‐year climatic fluctuation constitutes a source of variation that is rarely addressed alongside cultivar effects.

That the interaction was strongest in the pulp and juice, the fractions that constitute the edible portion of the berry, is of particular relevance to food‐quality assessment. These are the fractions in which season‐dependence of cultivar behavior was greatest and therefore the fractions for which single‐season data are least reliable. Conversely, the cultivar component of variance was concentrated in a small number of elements, principally zinc, iron, and chloride, for which cultivar identity remained the dominant influence irrespective of season; these elements may be the most tractable targets for genotype‐based selection. The dominance of the interaction term also motivates the treatment of compositional stability as a trait in its own right. The cultivars examined here differed by a factor of more than 1.6 in the extent to which their composition changed between seasons, from 1.75‐fold in Niagara to 2.87‐fold in Concord, and the ranking was not related in any obvious way to geographic origin, since the North American and Turkish‐bred cultivars were interspersed throughout it. That Niagara and Rizpem owed their stability principally to the pulp and juice fractions is potentially useful, since these are the fractions that determine the composition of juice and other processed products. We note, however, that stability estimated from two seasons at one site is a preliminary indication rather than an established varietal characteristic, and that confirming it would require multi‐environment trials over a longer period.

Interannual change was also directionally coherent for several elements in a way that the univariate cultivar comparisons alone would not reveal. Iron and zinc increased in every case in which they changed significantly, across all cultivars and all fractions, while manganese and phosphorus decreased in the large majority of significant comparisons. Rogiers et al. (2006) classified manganese and zinc as elements accumulating predominantly prior to *véraison* through xylem flow, whereas copper was considered conditionally phloem‐mobile; comparable element‐specific mobility patterns have been reported elsewhere, with restricted phloem transport of Mn and Zn and comparatively greater mobility of Cu in higher plants (Broadley et al. 2007; Palmer and Guerinot 2009; Hänsch and Mendel 2009; White and Broadley 2009; Marschner 2011). The opposite behavior of zinc and manganese between the two seasons observed here is not readily accommodated by a common xylem‐mediated mechanism and suggests that these elements responded to different seasonal drivers. Soil chemistry, redox status and rainfall distribution were not measured in this study, so the drivers themselves cannot be identified; we therefore report the pattern as an observed association and refrain from proposing a mechanism. The 7.1‐day delay in harvest in 2025 relative to 2024, at essentially unchanged soluble solids concentration, indicates that the second season was cooler or that ripening was otherwise slower, and it is consistent with the presence of a genuine seasonal signal. That soluble solids concentration itself explained none of the mineral variation, in any element or fraction, indicates further that the differences reported here are not an artifact of cultivars having been harvested at different physiological stages.

### 4.3 Elemental Associations and Multivariate Structure

Restricting the analysis of elemental associations to partial correlations, computed after cultivar, year and block effects had been removed, was intended to establish whether the associations reported in the grape mineral literature persist once these sources of variation are accounted for. They did: 186 of 312 pairs remained significantly correlated. The associations among the macronutrient group, in particular between phosphorus and potassium, potassium and calcium and nitrogen and calcium in the fleshy fractions, were the strongest recorded and were reproduced across fractions. These are statistical associations among concentrations measured in the same tissue and should not be read as evidence of shared transport pathways or of coordinated regulation; establishing that would require labeling or transport studies that the present design does not include. One feature of the correlation structure requires comment because it bears on the reliability of the underlying measurements rather than on grape physiology. In every fraction, copper, sodium and iron formed a group that was positively associated internally and negatively associated with the entire macronutrient group, the negative correlations between nitrogen and copper and between nitrogen and sodium being among the strongest in the study. The same opposition appeared independently in the principal component analysis, where the loading vectors for these three elements consistently opposed those of the macronutrients along the first axis. A biological explanation for such a coherent and fraction‐independent opposition is not evident. It is, however, exactly the pattern that would be produced if the determination of these particular elements were subject to a systematic influence that did not affect the others. Taken together with the copper concentrations that could not be reconciled with the management history of the vineyard (Section 3.2), this leads us to treat copper, and to a lesser extent sodium and iron, as analytically unverified in the present dataset. We report the observation rather than omit it, because it constitutes the clearest internal evidence bearing on the limitations described in the following section.

## 5 Limitations

Three limitations of the present study concern the analytical basis of the measurements and constrain the interpretation of absolute concentrations. First, method‐performance parameters were not systematically recorded for this dataset. Certified reference materials and procedural blanks were included in each analytical batch, but limits of detection and quantification, spike recovery, certified‐reference‐material recovery, and calibration linearity were not documented and cannot be reported retrospectively. The absolute concentrations presented here should accordingly be regarded as provisional and should not be compared directly with values reported in other studies. The relative comparisons on which every conclusion of this study rests, namely comparisons among cultivars and between seasons within a single fraction, are unaffected by a constant scaling factor and remain valid. Second, the juice fraction was digested directly, without prior drying, and the procedure used to convert the measured concentrations to a dry‐weight basis could not be documented. For this reason, no comparison is made between berry fractions anywhere in this study, and the hierarchical distribution of minerals among seed, skin, pulp, and juice, which would require a verified common basis of expression, is not addressed. All analyses reported here were conducted within fractions. Third, copper is reported but not interpreted. Copper concentrations reached 153.44 mg kg^−1^ and differed among cultivar × fraction × season combinations by more than four orders of magnitude, despite the absence of any copper‐containing application to the vineyard in either season, and copper participated in the anomalous correlation structure described in Section 4.3. In the absence of vineyard soil analysis or analytical validation data, the possibility of contamination or of a systematic analytical influence cannot be excluded, and no interpretation of copper is offered. The same reservation applies, to a lesser degree, to sodium and iron. Beyond these analytical constraints, the study was conducted at a single site over two growing seasons, and soil chemistry, rainfall distribution, and temperature were not recorded. Cultivar effects therefore cannot be separated from site‐specific effects, and the seasonal drivers of the interannual changes reported here cannot be identified. Mineral bioavailability, dietary contribution, processing behavior, and sensory quality were not assessed, so the results do not support conclusions about the nutritional value of these cultivars for human consumption. The stability ranking derived from two seasons should be regarded as a preliminary indication requiring multi‐environment confirmation.

## 6 Conclusions

This study quantified the sources of variation in the mineral composition of four berry fractions of eight *Vitis labrusca* cultivars over two growing seasons. The principal finding is that the year × cultivar interaction, and not cultivar or year individually, was the dominant source of variation, accounting on average for 39% of the total variance against 32% for cultivar and 14% for year, and being significant in 49 of 52 fraction × element combinations. The interaction was strongest in the pulp and juice, the fractions that constitute the edible portion of the berry. The practical consequence is that cultivar rankings for mineral concentration are season‐specific rather than fixed, as demonstrated by repeated reversals in which a cultivar occupied the highest position of the panel in one season and the lowest in the next. Single‐season mineral profiling of grapevine germplasm is therefore liable to be misleading, and multi‐year assessment should be regarded as a requirement rather than a refinement in studies of this kind. A second consequence is that the stability of mineral composition between seasons is a property that differs among cultivars and can be measured. In the present panel it varied by a factor of more than 1.6, from 1.75‐fold in Niagara to 2.87‐fold in Concord, and it was not related to the geographic origin of the cultivars. Where consistency of composition is desirable, as in processing, stability may deserve consideration alongside concentration itself. Zinc, iron, and chloride were the elements for which cultivar identity remained the dominant influence irrespective of season, and these are consequently the most promising candidates for genotype‐based selection within this panel. Interannual change was directionally coherent for several elements, iron and zinc increasing in every significant comparison and manganese and phosphorus decreasing in most, but the environmental drivers of these shifts were not measured and cannot be identified here. Because analytical validation data were unavailable and the basis of expression for the juice fraction could not be verified, the absolute concentrations reported here are provisional and no comparison between fractions is made. The conclusions above rest on within‐fraction comparisons, which are unaffected by these limitations. Confirming them will require multi‐site, multi‐season trials accompanied by soil and climatic monitoring and by full analytical validation.

## Author Contributions

**Besim Karabulut:** conceptualization, investigation, funding acquisition. **Yahya Uray:** conceptualization, methodology, validation, formal analysis. **Hüseyin Çelik:** conceptualization, investigation, formal analysis. **Bülent Köse:** software, methodology, conceptualization. **Cafer Kose:** conceptualization, methodology, data curation, formal analysis. **Metin Turan:** conceptualization, methodology, visualization, formal analysis. **Ozkan Kaya:** conceptualization, investigation, methodology, validation, writing – review and editing, writing – original draft, formal analysis, data curation, resources, software.

## Funding

The authors have nothing to report.

## Ethics Statement

This study involved only plant materials and did not include human participants or animal subjects; therefore, no ethical approval was required.

## Consent

All authors have read and approved the final manuscript and consent to its submission and publication.

## Conflicts of Interest

The authors declare no conflicts of interest.

## Supporting information


**Table S1:** Record of data verification and the resulting treatment of affected observations.
**Table S2:** Harvest dates and soluble solids concentration at harvest for each cultivar in the two seasons.
**Table S3:** Factorial analysis of variance of mineral concentrations within each berry fraction.

## Data Availability

The complete replicate‐level dataset analyzed in this study, comprising all 13 elements for four berry fractions of eight cultivars over two growing seasons, is provided as a Supporting Information. Tables S21–S3, containing the record of data verification, the harvest dates and soluble solids concentrations, and the full factorial analysis of variance, are provided as supplementary tables. Values identified as erroneous during verification are flagged rather than removed in the supplied dataset so that all decisions described in Section 2.4 can be reproduced.
